# Parasitic Infections and Associated Cognitive Outcome Among School‐Aged Children in Africa: A Systematic Review

**DOI:** 10.1002/brb3.71326

**Published:** 2026-03-30

**Authors:** Albertha Maku Adu, David Larbi Simpong, David Mawutor Donkor, Kwaku Sarfo Manu, Maxwell Hubert Antwi, Linus Kpelle, George Nkrumah Osei, Ansumana Bockarie

**Affiliations:** ^1^ Department of Medical Laboratory Science School of Allied Health Sciences University of Cape Coast Cape Coast Ghana; ^2^ Alzheimer's and Related Disorders Association of Ghana Tema Ghana; ^3^ Department of Medical Laboratory Science Faculty of Health Sciences Koforidua Technical University Koforidua Ghana; ^4^ Department of Internal Medicine and Therapeutics, School of Medical Sciences University of Cape Coast Cape Coast Ghana

**Keywords:** cognition, children, neurodevelopment, parasitic infections

## Abstract

**Background:**

Parasitic infections remain endemic among children, yet the neurocognitive and educational impact of these infections has not been systematically synthesized. This systematic review investigates the potential associations between parasitic infections and neurocognitive and educational outcomes among school‐aged children.

**Methods:**

A comprehensive review was conducted in accordance with PRISMA guidelines. Included studies encompassed both observational and interventional designs, examining cognitive and educational outcomes in relation to infections with *Schistosoma* spp., soil‐transmitted helminths (STHs), *Plasmodium* spp., and other common parasitic agents.

**Results:**

Of the 80 studies reviewed, 29 focused on *Schistosoma* spp., with 13 identifying significant cognitive impairments, particularly in memory, attention, and increased school absenteeism. STHs were investigated in 26 studies, 11 of which reported associations with reduced intelligence quotient (IQ), executive dysfunction, and poor academic performance. *Plasmodium* spp. was examined in 15 studies, with 9 highlighting cognitive deficits, especially postcerebral malaria. Additional parasitic infections, including tungiasis (3 studies), *Giardia* and *Entamoeba* spp. (4 studies), and *Taenia* spp. (2 studies), were consistently linked to adverse cognitive and educational outcomes.

**Conclusion:**

The most frequently implicated parasites are *Schistosoma* spp., STHs, and *Plasmodium* spp. demonstrate consistent associations with neurocognitive deficits. Pharmacological treatment alone may be insufficient; comprehensive strategies integrating early medical intervention, nutritional rehabilitation, and improved educational access are warranted. Future research should emphasize longitudinal methodologies, harmonized cognitive assessment tools, and cross‐sectoral approaches to mitigate these developments.

## Graphical abstract

Parasitic infections like schistosomiasis, soil‐transmitted helminths, and malaria can impair nutrient absorption, oxygen supply, and brain function, leading to memory deficits, attention deficits, reduced intelligence quotient, weak academic performance, and motor or executive deficits.

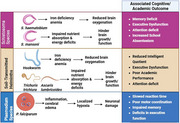

## 1 Introduction

Parasitic infections, particularly those caused by *Plasmodium* species, *Schistosoma* species, and Soil‐transmitted helminths (STHs), are the leading cause of disease burden in preschool and school‐aged children, contributing to thousands of morbidities every year (Alemayehu et al. 2025; Johnson et al. 2024; WHO 2023). These chronic endemic infections are common in sub‐Saharan Africa, South America, and Asia, and they contribute to inhibited social development and reduced quality of life in affected regions (Agrawal et al. 2024; Gasparotto et al. 2021). Their high burden is largely driven by environmental and socioeconomic conditions that favor parasite transmission, including poor sanitation, inadequate hygiene practices, and limited access to clean water (Moncayo Benalcázar 2018). Globally, over one billion individuals are affected by chronic soil‐transmitted helminth infections, while schistosomiasis impacts about 240 million individuals, with the majority of cases concentrated in low‐ and middle‐income countries (WHO 2023). In 2010, malaria and neglected tropical diseases (NTDs) were responsible for an estimated 6.4 million disability‐adjusted life years (DALYs) among school‐aged children in sub‐Saharan Africa, representing 16.5% of the total DALYs in this age group (Murray et al. 2012; EDITION 2013). The World Malaria Report (WHO 2023) highlights the disproportionate burden of malaria in Africa, where the Region accounts for approximately 95% of global cases and 96% of malaria‑related deaths. Schistosomiasis exhibits a similar epidemiological pattern, with more than 90% of cases concentrated in African populations (WHO 2023). This dual burden of parasitic disease is further intensified by the widespread occurrence of polyparasitism among African children, a condition that compounds the adverse effects on cognitive development and educational attainment (Ezeamama et al. 2018).

Though infection with parasites is rarely fatal, they are a significant source of morbidity (Verjee 2020; Craig and Scott 2014). In children, parasitic infections significantly affect nutritional status, physical growth, and overall health. These infections are associated with gastrointestinal blood loss, impaired nutrient absorption, reduced appetite, and anemia, which collectively contribute to stunting, malnutrition, and compromised cognitive development (Pullan et al. 2014). In addition to the well‐characterized physical morbidities, including anemia and organ damage, emerging evidence suggests that parasitic infection may exert a substantial negative impact on neurocognitive functioning, learning capacity, and academic achievement (Garrison et al. 2021; Sari et al. 2020). Consistent findings across studies reveal diminished attention, impaired memory, and reduced school attendance among affected children, highlighting the broader developmental and educational burden imposed by parasitic diseases in endemic regions (Kasambala et al. 2023; Carpio et al. 2016). Other studies have also highlighted that cerebral malaria infections during childhood can disrupt neurodevelopmental processes, with consequences that may persist into adulthood (Onaolapo and Onaolapo 2025). Affected individuals are at risk of long‑term neurological complications, reflecting the neurodegenerative outcomes of malaria (Rosa‐Gonçalves et al. 2022). Documented sequelae include motor deficits, movement disorders, ataxia, paresis, and seizure (Rosa‐Gonçalves et al. 2022). Cognitive impairments have been observed across a range of domains, including memory, attention, language processing, executive functioning, and motor coordination (Harvey 2019; Kasambala et al. 2023; Ezeamama et al. 2018). The biological mechanisms contributing to cognitive decline in chronic parasitic infection are complex and multifactorial. Persistent infection elicits systemic inflammation, marked by elevated levels of proinflammatory cytokines such as TNF‐α, IL‐6, and IFN‐γ, which are believed to impair neuronal function either by crossing the blood–brain barrier or via activation of neuroimmune signaling pathways (Carpio et al. 2016; Kasambala et al. 2023). Emerging evidence indicates that parasitic infections may produce neurological consequences even when overt clinical symptoms are absent. For example, subclinical *Plasmodium falciparum* parasitemia has been linked to measurable impairments in cognitive performance among school‑aged children (Johnson et al. 2024). More broadly, evidence from systematic reviews demonstrates that clinically silent infections sustain chronic low‑grade inflammation and cytokine dysregulation, mechanisms that contribute to subtle yet significant deficits in cognitive functioning (de Brito Duval et al. 2025; Idro et al. 2022). In parallel, parasite‐induced anemia and associated nutritional deficiencies adversely affect cerebral oxygenation and the availability of essential nutrients required for optimal brain development (Ezeamama et al. 2018). These converging pathophysiological processes lead to neuroinflammation and potential neurotoxicity, thereby hindering normal neurodevelopment (Kasambala et al. 2023). Existing evidence is largely based on prevalence studies, with relatively few investigations establishing a direct association between heavy helminthic infections in children and diminished performance in cognitive and educational assessments when compared with their uninfected or moderately infected peers (Ezeamama et al. 2018). The impact of schistosomiasis among school children on their academic achievement has also been documented (Donkoh et al. 2023), while cerebral malaria has been identified as a major factor contributing to persistent cognitive impairments (Idro et al. 2016).

This systematic review primarily focuses on African regions, reflecting the continent's disproportionate burden of parasitic infections among school‐aged children. The African context is prioritized because of distinctive epidemiological characteristics, including disproportionately high burden of polyparasitism, high rates of malaria co‐infection, pervasive malnutrition, limited healthcare infrastructure, and the limited availability of standardized cognitive assessment tools in African populations, all of which intensify the cognitive consequences of infection compared with other regions (Ayeh‐Kumi et al. 2025). While comparative evidence from Asia and South America provides a broader global context, the specific epidemiological patterns and health determinants in African settings highlight the importance of a region‐specific synthesis to inform targeted public health interventions (Mallewa and Wilmshurst 2014; Kihara et al. 2006). Additionally, integrating multiple parasitic diseases such as *Schistosoma* species and *Plasmodium* species will reflect the complex reality of polyparasitism in endemic regions, offering a comprehensive assessment of its cumulative impact on cognitive and educational outcomes. This work is undertaken with the view that early detection, together with an effective intervention strategy, can improve children's neurocognitive development and academic performance.

The review seeks to investigate two primary questions: (a) Among school‐aged children, is there evidence of a significant association between parasitic infections assessed through cross‐sectional or case‐control studies and reduced performance on neurocognitive tests or educational outcomes? (b) In prospective studies involving school‐aged children undergoing targeted treatment for parasitic infections, does the lack of treatment contribute to poorer results in neurocognitive assessments or academic achievement? This review specifically examines defined cognitive domains, addressing a gap in earlier reviews that primarily focused on overall cognitive performance or general intelligence measures, without detailed analysis of individual cognitive functions or consideration of educational outcomes such as school attendance. By targeting these overlooked aspects, the present review offers a more focused and comprehensive perspective. The central hypothesis posits that parasitic infections are closely associated with negative cognitive outcomes in school‐aged children living in endemic areas, underscoring the pressing need for comprehensive interventions that bridge the health and educational sectors. Parasitic diseases trigger the immune system, but they also impair cognitive functions.

## 2 Methodology

We described our findings and standard methodology using the Preferred Reporting Items for Systematic Reviews and Meta‐Analyses (PRISMA) guidelines. Before the search, the review was prospectively registered with the PROSPERO database (registration ID: CRD420250650953). The PICO framework, comprising Population, Intervention, Comparison, and Outcomes, was utilized to systematically define and structure the elements of the research question guiding this review.

### 2.1 Search Strategy

A comprehensive literature search was conducted using PubMed, Scopus, and Google Scholar. Filters were applied to include studies published from the 1940s up to 2025. Relevant publications were identified through the application of targeted keywords and Boolean operators. To enhance the breadth and depth of the review, an additional search of grey literature was undertaken. This included examining World Health Organization reports and academic theses, aiming to capture a more inclusive range of data beyond conventional academic platforms. Grey literature sources, particularly Google Scholar and designated institutional repositories, were also explored to minimize potential publication bias and ensure that the findings reflect a more holistic representation of existing knowledge.

A comprehensive description of the search strategy has been provided as Supporting Information material. Two investigators independently extracted individual data and assessed the titles, abstracts, and full texts. Any disagreements were resolved by a third person. If the third author alone could not resolve the issue, a consensus was reached among all investigators.

### 2.2 Eligibility and Selection Criteria

This systematic review specifically focused on school‐aged children between 5 years and above. All studies were incorporated into this review regardless of their design or publication year. This review included both interventional and observational studies that examined the relationship between cognitive function in school‐aged children using any psychometric test or assessed school (attendance, academic achievement) or neurocognitive outcomes, and infection with any parasitic species. Articles primarily focusing on preschool‐aged children were excluded due to the lack of educational measures and the use of neurocognitive tests, such as the Mullen test, which are challenging to categorize in terms of neurocognitive domains. Additionally, duplicates, meta‐analyses, studies not published in English, and primary studies on parasitic infections or soil‐transmitted helminth infections that did not measure cognitive outcomes were also excluded. Difficulties in accurately interpreting and critically evaluating studies due to language barriers, along with resource constraints that limited the translation of non‐English publications, were key factors contributing to the exclusion of studies published in languages other than English.

**FIGURE 1:**
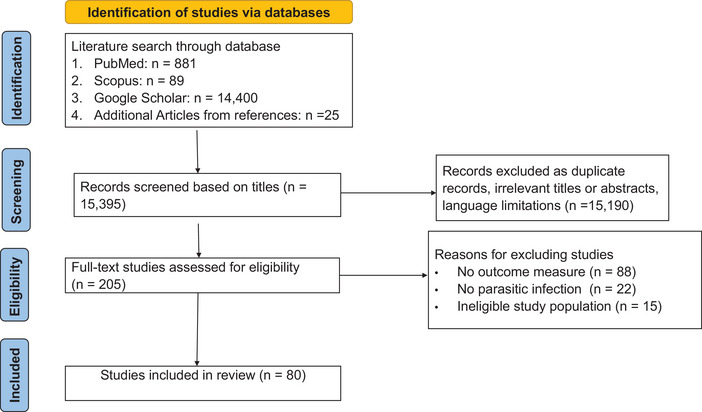
Flow diagram for search and selection of included studies.

The study selection procedure was systematically recorded using a PRISMA flow diagram, which outlined the rationale for exclusion at each stage of the review process (**Figure** **1**). All references incorporated into this systematic review were managed using Mendeley software (version 1.19.8). The software facilitated the organization of citations, identification and removal of duplicates, and classification of studies based on their eligibility status (included or excluded). Advanced functionalities, including automatic full‐text retrieval, were utilized to annotate references, monitor progress, and guide decision‐making throughout the review process, thereby ensuring a transparent and reproducible methodological workflow.

### 2.3 Data Extraction

The extracted information from each publication included the first author's name, year of publication, country of origin, parasite(s) involved, age range of subjects, study outcomes, study design, study features, subject features, and sample sizes. Subsequently, two hundred and five (205) full‐text articles were assessed for eligibility for inclusion in this review (**Table** **1**). Studies were excluded based on the following criteria: absence of reported outcome measures (*n* = 88), articles with cognitive outcomes attributed to conditions other than a parasitic infection (*n* = 22), and articles associated with preschool children (ineligible study population) (*n* = 15).

**TABLE 1:** Characteristics of included studies.

c	Author	Study design	Age group (years)	Outcome domain (s)	Cognitive Assessment tools	Parasitic Species Involved	Country	NOS Score	Key findings
1	(Musuva et al. 2017)	prospective cohort study	9–12	Academic achievement (Attention, learning problems)	BASC‐2 (Teacher Rating Scales)	*Schistosoma mansoni*	Western Kenya	4	Children with *Schistosoma mansoni* infection (*N* = 18) exhibited significantly higher scores for externalizing, internalizing, and school‐related behavioral problems compared with uninfected peers (*n* = 18). After administration of praziquantel, posttreatment evaluations (*n* = 35) revealed statistically significant enhancements in attention, learning capacity, and study‐related competencies (*p* < 0.05)
2	(Hürlimann Et Al. 2014)	Cross‐sectional survey/Longitudinal	5–14	Sustained attention, working memory	TEA‐Ch (the code transmission test), WISC‐IV (Digit Span Forward)	*Plasmodium falciparum*, *Plasmodium malariae*, Hookworm, *Trichuris trichiura*, *Ascaris lumbricoides*, *Entamoeba histolytica/* *E. dispar*, *S. mansoni*	Eastern Côte d'Ivoire	9	Children infected with helminths at baseline (*N* = 88) performed significantly better in sustained attention at follow‐up relative to their noninfected counterparts (*n* = 129; *p* < 0.05). However, infection status was not consistently associated with improvements in memory performance
3	(Jukes et al. 2002)	Cross‐sectional	9–14	Educational achievement, memory, and reaction times learning,	Fluency, DS, WF, CB, SWLT, Stroop, CRT, PBT, SS	*Schistosoma haematobium*	Tanzania	7	Children who are heavily infected with *Schistosoma haematobium* (*N* = 38) scored significantly lower than uninfected peers (*n* = 97) on verbal short‐term memory (digit span forwards: *p* = 0.003; backwards: *p* = 0.004) and reaction time tasks (silly sentences: *p* = 0.003; choice reaction time: *p* = 0.002). No significant differences were found in educational achievement. Cognitive impairment was most pronounced among infected children with stunted growth (HAZ < –2)
4	(King et al. 2020)	Longitudinal cohort studies	7–13	Academic difficulties, attention problems, and learning problems	BASC‐2 (Teacher Rating Scale)	*S. mansoni/S. haematobium*	Kenya and Tanzania.	7	Children infected with *S. mansoni* (*N* = 36) showed significant reductions in behavioral problems (externalizing, internalizing, school‐related) 3 weeks posttreatment; similar improvements were observed in egg‐negative peers (*n* = 36), indicating MDA benefits beyond detectable infection. Meta‐analysis of 30 studies confirmed that Schistosoma infection is associated with deficits in learning, memory, school attendance, and achievement
5	(de Clercq et al. 1998)	Cross‐sectional observational study	6–11	School performance, attendance	Teacher‐reported performance, Absenteeism rate	*S. haematobium*	South Western Bamako, Mali	7	Children infected with helminths (*N* = 251) demonstrated significantly lower academic achievement and increased school absenteeism compared with their uninfected counterparts (*n* = 286). Higher infection intensity was positively correlated with poorer academic grades (*p* < 0.005) and elevated absenteeism rates (*p* < 0.001), Absenteeism was the strongest predictor of academic decline, but infection remained a significant independent factor
6	(Nokes et al. 1999)	A double‐blind, randomized, placebo‐controlled trial	5–16	Working memory	WISC‐IV (Fluency, Digit‐Span Forwards), Corsi Block, Picture Search, and Free Recall	*S. japonicum*	Southwest Chengdu, China	9	Children treated for Schistosoma japonicum infection (*N* = 92) exhibited significant improvements in working memory tasks (fluency and Free Recall) relative to those in the placebo group (*n* = 89; *p* < 0.05), with the most pronounced cognitive gains observed among children aged 5–7 years. Enhancements in cognitive performance were particularly notable in children with stunted growth (HAZ <– 1.7) and low hemoglobin levels (< 11.7 g/dL). No significant differences were detected in Digit Span or Corsi Block performance
7	(Nazel et al. 1999)	Case‐control	9–12	Working memory, IQ, and scholastic achievement, School attendance	WISC, VFT, School records	*S. mansoni*	Kafr El Sheikh, Egypt	8	Children infected with helminths (*N* = 80) demonstrated significantly lower performance IQ and reduced scores in comprehension, vocabulary, and picture completion tasks (*p* < 0.05) compared with uninfected peers (*n* = 40). Notably, those with light‐intensity infections exhibited the poorest academic achievement and the highest rates of school absenteeism than both heavily infected and uninfected groups (*p* < 0.01)
8	(Meremikwu et al. 2000)	Cohort study	8.0–8.9	School performance, Attendance and aptitude tests.	Standardized teacher‐made test (comprehension, simple arithmetic, aptitude test), attendance rate	*S. haematobium*	South‐Eastern Nigeria	6	Among infected children (*N* = 145), repeated praziquantel administration was associated with a significant increase in academic pass rates, rising from 81.4% to 84.2% (*p* = 0.027), alongside a substantial reduction in infection prevalence from 69.1% to 21.6%. No significant improvement was observed in school attendance (*p* = 0.22)
9	(Inyang‐Etoh et al. 2018)	Cross sectional study	3–15	Academic performance	Classroom teacher reports	*S. haematobium*	Nigeria	7	Children infected with Schistosoma haematobium (*N* = 57) exhibited significantly lower academic performance, with the highest infection prevalence observed among those receiving ‘D & E’ grades (30%) compared with those scoring ‘F’ (10%) (*p* = 0.020). Infection was also significantly associated with hematuria and proteinuria (*p* ≤ 0.001)
10	(Tandoh et al. 2020)	Cross‐sectional	9–12	Executive functioning, IQ, and attendance	RCPM/school attendance record	*S. haematobium, S. mansoni*, soil‐transmitted helminth (STH)	Ghana	7	Children infected with helminths (*N* = 36) demonstrated a significantly higher prevalence of anemia compared with uninfected peers (*n* = 128; 69.4% vs. 48.4%; *p* = 0.03). However, infection status was not significantly associated with cognitive test outcomes (*p* = 0.68), and logistic regression analysis indicated that helminthiasis, anemia, and zinc deficiency were not significant predictors of cognitive performance
11	(Donkoh et al. 2023)	Cross‐sectional survey	5–16	Academic performance	Cumulative end‐of‐term examination results	*Strongyloides stercolari*, *Ancylostoma*, *A. lumbricoides*, *Taenia* spp., *E. vermicularis*, *T. trichiura* *S. stercolaris, Schistosoma* spp., *Entamoeba* spp., *Plasmodium* spp.	Ghana	7	Children infected with helminths (*N* = 63) exhibited significantly lower academic achievement compared with their uninfected counterparts (*n* = 54; *p* = 0.034). Infection status, in combination with weight‐for‐age z‐scores, significantly predicted academic performance (*R* ^2^ = 0.125); however, this effect was attenuated when adjusted for household income
12	(Onyirioha 2010)	Cross‐sectional study	primary school pupils	Academic performance, school attendance	Class teacher Score	*S. haematobium*	Niger Delta, Nigeria	5	Infected pupils (*N* = 1936) demonstrated lower mean academic scores (58.97%) and school attendance rates (81.91%) compared with uninfected peers (*n* = 1936), who recorded scores of 61.80% and attendance of 84.74%. Although these differences did not reach statistical significance (*p* > 0.05), psychological stress associated with infection may contribute to impaired learning outcomes.
13	(Epstein and Weisbrod 1974)	Cross‐sectional observational study.	13–14	School performance	Nelson Reading Skills Test (NRST) for grades 3–9 (Vocabulary, reading comprehension)	*S. mansoni*	Saint Lucia, Caribbean	7	Among infected children (*N* = 158), no statistically significant differences were observed in reading scores or overall academic performance (*p* > 0.05). Within a high‐prevalence rural sub‐sample (*N* = 267), Schistosoma infection alone was associated with reduced school absenteeism (*p* < 0.05), whereas co‐infection with Schistosoma and two additional parasitic infections was linked to increased absenteeism (*p* < 0.10). No significant effects were noted on anthropometric measures (height and weight) or class rank
14	(Loveridge et al. 1948)	Case‐control	11–18	Educational attainment	School test scores	*S. haematobium*	Zimbabwe	5	Among European boys (*N* = 199), *S. mansoni* infection was significantly associated with lower academic placement (*p* < 0.01). In contrast, among African pupils (*N* = 365), infected children performed comparably or better than their uninfected counterparts, with 77% of top‐ranked students testing positive for schistosomiasis. No adverse impact of infection on academic performance was observed in this cohort
15	(Ezeamama et al. 2005)	Cross‐sectional study	7–18	Learning and memory cognitive	WRAML/VF/ PNIT Learning (VL, AL, VLL) verbal memory (SM, STM, NLM),	*A. lumbricoides, S. japonicum, Necator americanus, and T. trichiura*	Philippines	7	Among infected children (*N* = 319), distinct helminth‐specific cognitive impairments were identified: Schistosoma japonicum was associated with reduced learning ability (OR = 3.04), Ascaris lumbricoides with impaired memory function (OR = 2.2), and *T. trichiura* with diminished verbal fluency (OR = 4.5). No significant effects were observed on nonverbal intelligence. These associations persisted after controlling for socioeconomic status, nutritional status, and hemoglobin levels
16	(El‐Masry et al. 2007)	Cross‐sectional	6–16	Scholastic achievement	First‐term examination report	*Entamoeba histolytica, E. vermicularis, Giardia lamblia*, *A. lumbricoides, Ancylostoma duodenale*, *T. trichiura*, *S. haematobium*	Egypt	7	Children infected with helminths (*N* = 370) exhibited significantly poorer academic performance, with 34.3% scoring below 50% compared with 21.9% of uninfected peers (*n* = 370; *p* = 0.000). They also showed higher rates of school absenteeism (≥4 days/month in 58.9% vs. 47.6%; *p* < 0.05), anemia (52.4% vs. 32.7%; *p* = 0.000), and stunting (31.6% vs. 18.7%; *p* = 0.000). Key risk factors contributing to these outcomes included inadequate hygiene practices, low socioeconomic status, and delays in receiving treatment
17	(Olsen 1998)	Cross‐sectional community survey	6–15	School enrolment rates	Report from parents and children during the house‐to‐house survey	Hookworm, *A. lumbricoides*, *T. trichiura*, *S. mansoni*	Kisumu District, Western Kenya	5	Nonenrolled children contributed >50% of infections and up to 85% of egg output, limiting community‐level impact
18	(Nyamwange 2012)	Pre‐and post‐double‐blind placebo‐controlled trial	7–11	Academic Achievement, Absenteeism, Intellectual maturity	VF, SA, DSF, FR, DSB, PS, CD, DAP, School records.	*S. mansoni*	Kenya	7	Among children infected with *S. mansoni* (*N* = 121), treatment was associated with marked cognitive improvements over 6 months, with gains observed in Free Recall (64.7%) and Coding (88.2%) tasks. These effects were more pronounced in those with severe baseline infection compared with mildly infected counterparts (Free Recall: 57.6%; Coding: 66.7%). In contrast, uninfected controls (*n* = 34) demonstrated no significant cognitive change. Posttreatment, school absenteeism showed modest improvement, and pre‐existing cognitive disparities between infected and uninfected groups were substantially reduced
19	(Ezeamama et al. 2012)	Nested prospective cohort study	7–19	Executive function, learning and working memory	PNIT, VF, WRAML (Learning, Verbal memory)	*S. japonicum*	Philippines	9	Among children infected with Schistosoma japonicum (*N* = 253), those who remained infection‐free for at least 12 months following praziquantel treatment (*n* = 25) demonstrated notable improvements in verbal memory and fluency. Substantial gains on the WRAML were observed in children exhibiting reductions in two or more soil‐transmitted helminth (STH) species (*n* = 29). Baseline polyparasitism with multiple STH species (*N* = 119) was associated with significantly lower scores on the PNIT
20	(Bell et al. 1973)	Quasi‐experimental design	8(Epstein‐12	Executive function, learning and working memory	PNIT, VF, WRAML (Learning, Verbal memory)	*S. japonicum*	Philippines	9	Children infected with *S. mansoni* (*N* = 134) exhibited significantly lower performance on Raven's Progressive Matrices compared with uninfected peers (*n* = 220; *p* < 0.01). Following treatment with hycanthone, a subset of infected children (*n* = 58) demonstrated cognitive improvements that aligned with scores observed in uninfected controls. No statistically significant differences were noted in academic examination results or anthropometric growth measures; however, modest posttreatment improvements were evident
21	(Ejezie and Ade‐Serrano 1981)	Cross‐sectional study design	6–15	Attendance, School performance	Average grades scored, School attendance records	*S. haematobium*	Nigeria	5	In a cohort of 681 Nigerian schoolchildren, *S. haematobium* prevalence was 24%, with a mean egg count of 435 per 10 mL of urine. Clinical manifestations such as haematuria and proteinuria were frequently observed; however, no significant associations were identified between infection intensity and school attendance or academic performance. The authors suggest that the limited morbidity and negligible educational impact may reflect low transmission intensity and partial immunity acquired through prior exposure
22	(Jordan and Randall 1962)	Cohort	12–19	Scholastic Achievement	Class Rank Improvement	*S. haematobium*	Tanzania	5	In a cohort of 141 Tanzanian schoolboys, the prevalence of *S. haematobium* and *S. mansoni* was 37.5% and 42.5%, respectively. *S. mansoni* infection was associated with lower haemoglobin levels and impaired growth, whereas S. haematobium had minimal impact on growth metrics, although treatment was linked to subsequent improvements. Educational outcomes were heterogeneous; some infected boys demonstrated superior academic performance relative to their peers before treatment and exhibited further gains following intervention
23	(Ekanem et al. 1994)	Cross‐sectional	5–15	Academic achievement, attendance	Teacher‐given test, % attendance	*S. haematobium*	Nigeria	4	A cross‐sectional study of 510 Nigerian schoolchildren aged 5 to 15 years reported a *S. haematobium* prevalence of 44%, with peak infection rates observed among children aged 10 to 15 years. The majority of infections were classified as light intensity, with 73% of cases presenting 1–49 ova per 10 mL of urine. No significant differences were identified between infected and uninfected children in terms of anthropometric indicators, school attendance, or academic performance. The authors suggest that the absence of measurable impact may be attributable to the low parasitic burden and potential regional variation in parasite strain virulence
24	(Castle et al. 1974)	Cross‐sectional	11–18	Achievement, Learning, Memory	The Thurstone PMA test (VRSNW)	*S. haematobium*	South Africa	4	Confirmed cases (*n* = 26) demonstrated significantly reduced Vocabulary (V) and Reasoning (R) scores (*p* < 0.01), with differences primarily attributed to socioeconomic status. Although Similarities (S) and Number (N) scores were also lower, these did not reach statistical significance. Among treated individuals (*n* = 9), posttreatment improvements in S and N scores suggest that cognitive fatigue associated with infection may be reversible
25	(Alemayehu et al. 2025)	Cross‐sectional	5–14	academic achievements	Record from student rosters	*A. lumbricoides*, Hookworm*, T. trichiura*	Gesha woreda, southwest Ethiopia	7	The overall prevalence of soil‐transmitted helminth (STH) infections was 36.7% among the study population (*N* = 493). Infected children exhibited significantly lower academic performance, with 42% achieving passing scores compared with a higher proportion among uninfected peers (*p* < 0.05). The predominant helminth species identified were *Ascaris lumbricoides* (20.1%), *T. trichiura* (10.1%), and hookworm (6.5%)
26	(Bangirana et al. 2011)	Case‐control	5–12	Memory, learning, visual spatial skills, attention academic achievement	KABC‐II, TOVA, WRAT‐3, MC‐HOME	*P. falciparum*	Uganda	6	Children diagnosed with malaria accompanied by neurological involvement (*N* = 62) exhibited significantly higher levels of internalizing behavioural problems compared with matched controls (*n* = 61; *p* = 0.007). Effects on attentional function were marginal (*p* = 0.05), and no statistically significant differences were observed across other cognitive or academic domains at the 3‐month follow‐up
27	(Kariuki et al. 2014)	Case‐control	6–9	working memory, attention, executive function,	SOPT, CNT, TEA‐Ch	*P. falciparum*	Kenya	8	Children experiencing complex seizures secondary to severe malaria (*N* = 58) demonstrated significantly impaired executive functioning relative to unexposed controls (*n* = 56), as evidenced by reduced vigilance (*p* = 0.030), increased errors on the Self‐Ordered Pointing Task (SOPT; *p* = 0.029), and diminished everyday attentional capacity (*p* = 0.019).These associations remained significant after adjusting for age, sex, schooling, maternal education, and perinatal history. No comparable deficits were observed among children with other neurological phenotypes
28	(Elson et al. 2023)	Cross‐sectional survey	8–14	School attendance and academic achievement	Exam scores recorded	*Tunga penetrans*	Kenya	7	In Kenyan schoolchildren, tungiasis was associated with increased absenteeism, lower academic performance in core subjects, and substantial physical morbidity, including severe pain and diminished quality of life. Cognitive disruption appeared to be mediated by the infection's physical symptomatology. The authors advocate for the inclusion of tungiasis within school‐based neglected tropical disease (NTD) control programs to protect learning and developmental outcomes
29	(Donkoh et al. 2022)	Cross‐sectional survey	5–16	Academic performance	cumulative end‐of‐term examination results	*Taenia* spp., *S. heamatobuim*, *A. lumbricoides, Ancyclostoma duodenale, T. trichiura, Plasmodium* spp.	Ghana	5	Children infected with intestinal parasites (*N* = 63) demonstrated significantly lower academic performance (mean score: 53.7 ± 11.5%) compared with uninfected peers (*n* = 54; 59.6 ± 16.9%; *p* = 0.034). Helminth infection was significantly associated with increased prevalence of anaemia (65.8% vs. 42.1%; *p* < 0.001), although nutritional status, assessed by weight‐for‐age, did not independently predict academic outcomes (*p* = 0.117). Multivariate regression indicated that both infection status and nutritional status jointly contributed to academic performance (*R* ^2^ = 0.125; *p* < 0.001); however, the predictive effect of infection was attenuated upon inclusion of household income in the model (*R* ^2^ = 0.164; *p* = 0.102)
30	(Opeyemi Olopade et al. 2018)	Cross‐sectional	6–12	IQ	Draw‐a‐person‐test	*A. lumbricoides*, Hookworm*, Hymenolepis nana*	Nigeria	7	Parasitic infections were present in 24% of pupils, with *Ascaris lumbricoides* accounting for the majority (22.1%). No statistically significant associations were observed between infection status and cognitive performance (*p* = 0.145), nor with nutritional status as assessed by BMI (*p* = 0.672). Regular handwashing was significantly protective against infection (*p* = 0.035), whereas defecation in bushes or refuse dumps was associated with increased infection risk (*p* = 0.032).
31	(Orish et al. 2018)	Cross‐sectional	6–14	School performance	Arithmetic test	*P. falciparum*	Ghana	7	Children with asymptomatic Plasmodium falciparum infection (*N* = 305) exhibited markedly higher arithmetic failure rates compared with uninfected peers (62.8% vs. 37.2%; *p* = 0.001), alongside significantly lower mean scores (47.35%; *p* = 0.021). In multivariate analysis, RDT‐confirmed malaria remained an independent predictor of poor academic performance (adjusted odds ratio [AOR] = 1.92; *p* = 0.003). Anaemia showed an initial association with poor performance (*p* = 0.03), but this effect was attenuated after adjustment. No significant associations were observed with socioeconomic variables
32	(Clarke et al. 2017)	Cluster‐randomized trial	9–12	Sustained attention, visual search, numeracy, vocabulary, and writing	TEA‐Ch, vocabulary test in French, writing and numeracy skills, and a visual search task	*P. falciparum*	Mali	9	Implementation of integrated parasite control (IPC) interventions across 40 schools significantly reduced parasitaemia (odds ratio [OR] = 0.005; *p* < 0.001) and anaemia prevalence (OR = 0.56; *p* = 0.001) among children aged 9–12 years. IPCs were also associated with improved sustained attention, reflected by a positive shift in standardized scores (z‐score = +0.23; *p* < 0.001). No statistically significant effects were observed across other assessed cognitive domains
33	(Melesse et al. 2017)	Cross‐sectional study	7–19	School performance	Academic records (student's grade report, grade averages, absenteeism	*A. lumbricoides*, hookworm*, G. lamblia*	Ethiopia	5	Children with intestinal parasitic infection (IPI; *N* = 269) were significantly less likely to achieve above‐average academic scores compared with uninfected peers (15.6% vs. 74.2%; *p* < 0.001). Nutritional deficits, including anaemia, stunting, and thinness, were also associated with poor academic performance (all *p* < 0.01). In multivariate analysis, IPI remained a strong independent predictor of low academic achievement (adjusted odds ratio [AOR] = 0.02; *p* < 0.001)
34	(Grigorenko et al. 2006)	Double‐blind, randomized controlled trial with a three‐group design	11–13	Cognitive abilities, Academic achievement, short‐term memory, selective attention, and retrieval from semantic memory, motor functioning, and reaction time	Reading, spelling, arithmetic, Dynamic Tests, Static Tests	*S. haematobium*, Hookworm	Tanzania	9	Children treated for moderate to heavy helminth infections (*N* = 92) demonstrated superior gains in dynamic cognitive tasks, including Syllogisms and Sorting, relative to untreated peers (*N* = 94), with performance comparable to uninfected controls (*n* = 68). While static test scores improved across all groups over time, only the Spanish Word‐Learning task showed sensitivity to treatment status. No significant differences were observed between groups in academic achievement outcomes
35	(Perignon et al. 2014)	Cross‐sectional observational study	6–16	Executive functions, attention, intelligence	RCPM, WISC III (block design, picture completion)	STH	Cambodia	7	Severe stunting and iron‐deficiency anemia, particularly among boys, were significantly associated with lower performance on cognitive assessments, including Raven's Coloured Progressive Matrices (RCPM), Picture Completion, and Block Design. Among girls, parasitic infection was linked to cognitive impairment and elevated risk of poor test outcomes. Marginal iron status also correlated with reduced cognitive scores. Socioeconomic status (SES) influenced RCPM performance but did not confound the observed associations between nutritional deficits and cognitive outcomes
36	(Gall et al. 2017)	Cross‐sectional observational study	8–12	selective attention, academic achievement	d2‐test, End‐of‐year school results	*T. trichiura, A. lumbricoides*	South Africa	7	Children infected with soil‐transmitted helminths (STH) (*N* = 259) demonstrated significantly lower selective attention, characterized by reduced correct positive responses and increased error rates, compared with their uninfected counterparts. Infected children also exhibited lower academic performance (mean difference: –0.45 SD) and diminished grip strength (mean difference: –1.2 kg). Maximal oxygen uptake (VO_2_ max) and socioeconomic status (SES) were positively associated with cognitive outcomes. While stunting was significantly correlated with reduced grip strength, it did not independently predict cognitive performance following statistical adjustment
37	(Liu et al. 2015)	Cross‐sectional study	9–11	Working memory index, processing speed index, absenteeism, and performance	WISC‐IV, school records, TIMSS	*A. lumbricoides, T. trichuria, A. duodenale or Necator americanus*	China	7	42% of children were infected with soil‐transmitted helminths (STH), and this group exhibited significantly lower cognitive and anthropometric outcomes compared with uninfected peers. Specifically, infected children had reduced scores on the working memory index (WMI: –3.4) and processing speed index (PSI: –4.7), as well as lower height‐for‐age (HAZ: –0.34), weight‐for‐age (WAZ: –0.28), and mathematics achievement (–8.9 points). Among STH types, *T. trichiura* infection was associated with more pronounced deficits than Ascaris lumbricoides. Although school absenteeism was higher among infected children, the association was not statistically significant after adjustment for confounders
38	(Beckmann et al. 2021)	Cross‐sectional	5–12	Academic achievement, accuracy, reaction time	end of year results, Flanker task,	*A. lumbricoides, T. trichiura, and/or A. duodenale and Necator americanus*	South Africa	7	In female participants, stunting, inadequate dietary diversity, and household food insecurity were significantly associated with reduced academic performance and slower reaction times on the Flanker task. Additionally, soil‐transmitted helminth (STH) infection was linked to diminished Flanker task accuracy. No statistically significant associations were observed among male participants. Socioeconomic status (SES) and hemoglobin levels did not independently predict cognitive or academic outcomes in either sex
39	(Degarege et al. 2022)	Cross‐sectional study	6–16	Academic performance	Mean academic semester score	*S. mansoni, A. lumbricoides, Hookworms, Hymenolepis. nana, E. vermicularis, Taenia* spp*., T. trichiura*	Ethiopia	6	Among the 532 children identified with helminth infections, mean hemoglobin levels were significantly lower by 0.16 g/dL compared with their uninfected counterparts, accompanied by a markedly increased risk of anemia (adjusted odds ratio [aOR] = 1.90). Helminth‐infected children also demonstrated reduced linear growth, reflected in a mean decrement of 0.15 in height‐for‐age z‐scores (HAZ). Academic performance was adversely affected, with infected children scoring an average of 66.4%, compared with 69.6% among uninfected peers (*β* = –2.22). The most substantial impairments were observed in children infected with *S. mansoni* and those with polyparasitic infections
40	(Morad and Allam 2018)	Cross‐sectional study	6–12	I.Q., School achievement, school attendance	Stanford‐Binet intelligence scale, grade score, and Attendance record book	*G. lamblia* *E. histolytica* *E. vermicularis* *H. nana* *A. lumbricoides* *S. mansoni* *T. trichiura*	Egypt	7	Among children with parasitic infections (*N* = 668), body weight was significantly lower by 2.3 kg relative to uninfected peers. The prevalence of anemia was elevated at 59.5%, and cognitive impairment was notable, with 48.3% of infected children scoring below average on standardized IQ assessments. Academic achievement was also adversely affected, with a higher proportion of infected children scoring below 50% on school assessments (9.8% vs. 5.6%). The most pronounced deficits were observed in cases of mixed‐species infections and among children infected with hookworm
41	(Chandy et al. 2009)	Prospective cohort study	5–12	Working memory, executive attention, tactile‐based learning	KABC‐II, TOVA, TPT	*P. falciparum*	Uganda	9	Children with a history of cerebral malaria (*N* = 38) exhibited a 3.67‐fold increased likelihood of cognitive deficits at two‐year follow‐up compared with peers without cerebral involvement (26.3% vs. 7.6%). Deficits were particularly pronounced in attentional domains, affecting 18.4% of cases. Notably, impairments either persisted or emerged beyond 6 months postinfection. In contrast, no significant cognitive impairment was observed among children with uncomplicated malaria
42	(Johnson et al. 2024)	Cross‐sectional study	7–14	attention, cognitive flexibility, episodic memory, processing speed, and working memory	DCCS, Flanker, PSM, PC, LS	*P. falciparum*	Kenya	7	Among children with asymptomatic Plasmodium falciparum parasitemia (*N* = 156), cognitive performance was significantly reduced, primarily through tumor necrosis factor (TNF)‐mediated inflammatory pathways (*β* = –0.25). TNF levels independently predicted lower cognitive scores (*β* = –0.022) and accounted for approximately 29% of the total effect of parasitemia on cognitive outcomes. Notably, parasitemia status alone did not exert a direct effect on cognition in the absence of elevated inflammatory markers
43	(Nsanzimana et al. 2019)	Descriptive cross‐sectional study	6–18	School attendance, school performance	attendance register, trimester average scores	*T. penetrans*	Rwanda	7	Tungiasis was identified in 87 children, representing a prevalence of 23%. The condition was strongly associated with indicators of poor hygiene, including visibly dirty feet and clothing, as well as behavioral and environmental risk factors such as walking barefoot (adjusted odds ratio [AOR] = 78.41) and residing in homes with earthen floors (AOR = 28.79). Educational consequences were notable, with affected children exhibiting significantly higher odds of school absenteeism (AOR = 19.16) and poor academic performance, defined as scoring below 50% on standardized assessments (AOR = 110.85).
44	(Gerber et al. 2021)	Cross‐sectional study	6–12	Attention, reaction time, Performance, and academic achievement	Flanker task, mean accuracy, mean reaction time, end‐of‐year results	*A. lumbricoides*, *T. trichiura*, Hookworms	South Africa	7	Among girls, higher cardiorespiratory fitness (CRF) was associated with improved school grades and greater accuracy on the Flanker task, whereas soil‐transmitted helminth (STH) infection was linked to reduced inhibitory control (β = –0.17). In boys, socioeconomic status (SES) and grip strength were positively associated with academic performance and inhibitory control. Stunting was consistently associated with impaired cognitive and academic outcomes across both sexes
45	(Brasil et al. 2017)	Cross‐sectional study	2–10	IQ	WISC‐III	*Plasmodium vivax*	Brasil	6	Children with a history of four or more prior episodes of Plasmodium vivax malaria demonstrated significantly reduced verbal intelligence quotient (VIQ; mean difference = –13, *p* = 0.005), full‐scale IQ (FIQ; mean difference = –9, *p* = 0.042), and verbal comprehension index (mean difference = –10, *p* = 0.0382) compared with uninfected controls. No significant differences were observed in performance IQ (PIQ) or other factorial indices. The observed verbal deficits are plausibly attributable to cumulative school absenteeism and limited parental educational attainment, which may constrain environmental stimulation critical for language development
46	(Otieno et al. 2023)	cross‐sectional study	8–14	Attention, memory, language, perceptual‐motor, executive function, mental health outcomes, school absenteeism	EGRA, CFT, CTMT, SCWT, backward digit span task, bead threading test, CBCL, School register	*T. penetrans*	Kenya/Uganda	7	Tungiasis was associated with domain‐specific cognitive and academic impairments. Mild infection correlated with reduced literacy scores (mean difference = –8.9), language abilities (–1.7), cognitive flexibility (–6.1), and working memory (–0.3). Children with severe infection exhibited more pronounced deficits in literacy (–11.0), response inhibition (–2.2), fine motor coordination (–0.7), and numeracy (–3.0). No statistically significant associations were observed between tungiasis and mental health outcomes after adjustment. The observed impairments are likely mediated by infection‐related pain, social stigma, and school absenteeism
47	(Jumbe and Siwila 2024)	Cross‐sectional study	8–12	Working memory, processing speed, and academic performance	school records of scores obtained by children	*A. lumbricoides*, hookworm*, Taenia* spp., *S. mansoni, Strongyloides stercoralis*	Zambia	5	In a cross‐sectional study of 209 Zambian schoolchildren, the prevalence of soil‐transmitted helminth (STH) infection was 4.8%, with higher rates observed among males and younger children. All infections were of light intensity. No significant associations were found between STH infection and body mass index (BMI), working memory, processing speed, or academic achievement. The authors recommend larger‐scale studies to more robustly evaluate potential cognitive and educational effects
48	(Said et al. 2018)	Descriptive cross‐sectional study	6–12	School achievement	Student Performance Assessment Form	Intestinal parasites	Egypt	5	Among Egyptian schoolchildren, intestinal parasitic infections were significantly associated with reduced cognitive and behavioral performance. Infected children scored lower in motivation (mean difference = –4.1), concentration (–11.1), creativity (–4.2), and overall academic performance (–28.6; *p* < 0.0001) compared with uninfected peers. These deficits were primarily attributed to underlying anemia, increased school absenteeism, and inadequate hygiene practices
49	(Gardner et al. 1996)	Randomized, double‐blind, placebo‐controlled trial	Primary school children	Learning, working memory, verbal information processing, and visual information processing	French‐learning; digit spans (forwards and backwards), Corsi block span; fluency; picture search; silly sentences	*T. trichiura*	Jamaica	9	In Jamaican children with light to moderate *T. trichiura* infection (≥1200 eggs per gram), cognitive impairment was limited to performance on the Silly Sentence Test (*p* < 0.001), with no significant deficits observed across other assessed domains. Anthelmintic treatment did not yield measurable cognitive benefits. Findings suggest that the neurocognitive impact of Trichuris infection may be minimal in well‐nourished pediatric populations
50	(Hoffman et al. 2008)	Cross‐sectional study	6–11	fluid intelligence (reasoning), short‐term auditory attention, and memory	Raven test, WISC‐III,	Hookworm, *A. lumbricoides*, *S. mansoni*	Brazil	7	Moderate to high‐intensity Ancylostoma (hookworm) infection was significantly associated with impaired performance on the Coding subtest (odds ratio [OR] = 3.20), while moderate Ascaris lumbricoides infection predicted lower scores on Raven's Progressive Matrices (OR = 2.03). Co‐infection with both helminths was linked to markedly reduced Arithmetic performance (OR = 6.67), indicating a compounded cognitive burden attributable to polyparasitism
51	(Halliday et al. 2012)	cross‐sectional	5–18	Sustained attention	TEA‐Ch, Raven's Progressive Matrices task	*P. falciparum*	Kenya	7	A baseline cross‐sectional study conducted among 2,400 school‐aged children (5–18 years) across 51 schools in Kenya reported a Plasmodium falciparum infection prevalence of 13%, with a notable association between infection and anaemia (overall prevalence: 45.5%). High parasite densities were significantly linked to increased anaemia risk (adjusted odds ratio [AOR] = 3.68; 95% confidence interval [CI]: 2.12–6.38). Despite this, neither malaria infection nor anaemia status was predictive of performance on standardized cognitive and educational assessments, including measures of attention, reasoning, literacy, and numeracy. Educational performance was more strongly determined by socioeconomic status, parental education levels, and the quality of classroom infrastructure
52	(Nankabirwa et al. 2013)	Cross‐sectional study	6–14	Sustained attention, abstract reasoning	Code transmission test, Ravens matrices	Plasmodium specie	Uganda	7	Among Ugandan children, asymptomatic Plasmodium infection, present in approximately 30% of the cohort was associated with significantly lower performance in abstract reasoning (mean difference: –0.6) and sustained attention (mean difference: –1.6) compared with uninfected peers. Notably, attention scores demonstrated a negative correlation with increasing parasite density. Independent of malaria status, Ascaris infection was also linked to reduced abstract reasoning scores (mean difference: –1.4). No significant cognitive impact was observed in relation to anemia. Socioeconomic status (SES) was positively associated with abstract reasoning outcomes but showed no relationship with sustained attention
53	(Kuong et al. 2016)	Cross‐sectional study	6–16	intellectual ability, executive functions, visual discrimination, attention	RCPM, block design, picture completion,	Hookworm*, A. lumbricoides, Taenia* spp., *Fasciola, T. trichiura*	Cambodia	7	Children infected with hookworm demonstrated significantly lower cognitive performance across multiple domains, including picture completion (mean difference: –0.65), Raven's Progressive Matrices (–0.78), and block design tasks (–2.03). These cognitive impairments were primarily mediated by reduced body iron stores, with infection intensity showing a strong correlation with iron depletion. No significant associations were observed between cognitive outcomes and levels of vitamin A, zinc, markers of inflammation, or anthropometric indicators. Notably, hookworm infection emerged as a significant risk factor for anemia only when body iron was excluded from the analytical model, underscoring the mediating role of iron status in the infection‐anemia pathway
54	(Watkins et al. 1996)	Double‐blind, placebo‐controlled randomized trial	7–12	School performance (vocabulary, reading, Peabody Picture Vocabulary Test), attendance	IVT, IRT, PPVT teachers’ attendance books	*A. lumbricoides*, *T. trichiura*	Guatemala	9	In a randomized controlled trial involving 246 Guatemalan children, albendazole effectively eliminated Ascaris lumbricoides infections but demonstrated limited efficacy against *T. trichiura*. After 6 months of treatment, no significant improvements were observed in reading ability, receptive vocabulary, Peabody Picture Vocabulary Test (PPVT) scores, or school attendance. Vocabulary outcomes marginally favored the placebo group (*p* = 0.057), though this did not reach conventional levels of statistical significance. While correlations indicated modest associations between helminth burden and reduced cognitive scores, the intervention itself did not yield measurable cognitive or educational benefits. Instead, school performance was more strongly associated with nonbiological factors, including gender, school quality, and primary language spoken at home
55	(Raj et al. 1997)	prospective, controlled pre–post intervention design.	Primary school children	School attendance	School registers	*A. lumbricoides, T. trichiura*, Hookworm	Northeastern Malaysia	7	In a nonrandomized intervention study involving 249 Malaysian schoolchildren, administration of albendazole led to a reduction in the prevalence of Ascaris lumbricoides and *T. trichiura*. Ascaris infection was associated with increased school absenteeism, with infected children missing an average of 3 days compared with 1 day among uninfected peers (*p* = 0.01). However, posttreatment attendance rates did not differ significantly from those of uninfected children, suggesting a limited impact of deworming on school participation. Correlational analyses revealed weak associations between helminth burden and absenteeism, and no evidence was found to support a causal relationship between helminth infection and school attendance
56	(Sari et al. 2012)	Randomized, open‐label, placebo‐controlled trial	Primary‐school age	Working memory, processing speed, verbal understanding, and IQ	WISC	*A. lumbricoides*, *T. trichiura*, Hookworm	Indonesia	7	In a randomized controlled trial involving 112 Indonesian children with mild soil‐transmitted helminth (STH) infections, albendazole administration over 3 months resulted in statistically significant improvements in cognitive performance compared with placebo. Specifically, treated children demonstrated gains in digit span scores (+1.1; *p* = 0.024) and total IQ (+4.9 points; *p* = 0.027). Although other cognitive subtests exhibited trends favoring the intervention, these did not reach statistical significance. Nutritional status was comparable between groups, with approximately 27% of participants classified as malnourished
57	(Tiruneh et al. 2001)	Cross‐sectional study	6–15	School attendance, absenteeism, and rates of school dropout	% Enrolled, school records.	*S. mansoni*	Ethiopia	7	A study of 1200 children revealed significantly higher *S. mansoni* prevalence and infection intensity among school attenders compared with nonattenders. Prevalence ranged from 67% to 85% in attenders versus 44% to 61% in nonattenders (*p* < 0.01), while infection intensity (measured in eggs per gram, epg) was higher among attenders (135–177 epg) than nonattenders (100–166 epg), with statistically significant differences observed only in the Gorgora region (*p* < 0.05). After adjusting for confounders, school attendance was associated with increased odds of infection (OR = 2.32; 95% CI: 1.77–3.05). The authors advocate for school‐based deworming interventions while emphasizing the need to address infection risks among nonattending children
58	(Vitor‐silva et al. 2009)	Prospective cohort study	5–14	School performance, school absenteeism	Class teacher's evaluation, teacher's attendance record,	*P. vivax*, *P. falciparum*	Brazil	9	Over the follow‐up period, 70 children (35.4%) experienced at least one episode of malaria, with Plasmodium vivax accounting for the majority of cases (69.2%), followed by *P. falciparum* (25.5%) and mixed infections (5.3%). Multivariate logistic regression analysis, adjusted for age, maternal education, school absenteeism, and duration of residence, identified malaria infection as an independent predictor of poor school performance (OR = 1.91; 95% CI: 1.04–3.54; *p* = 0.039). Neither nutritional status nor anaemia demonstrated significant associations with academic outcomes. The authors conclude that even nonsevere malaria episodes may adversely affect cognitive development and educational attainment, thereby contributing to the persistence of socioeconomic underdevelopment in endemic regions
59	(Simeon et al. 1995)	Randomized, double‐blind, placebo‐controlled trial	6–12	School achievement, School attendance	WRAT, school registers	*T. trichiura*	Jamaica	9	No significant overall treatment effects were observed on academic achievement, school attendance, or growth parameters. However, subgroup analyses revealed differential responses: children with heavy infection demonstrated marginal improvements in spelling scores (*p* = 0.06); those with light infection showed a significant increase in body mass index (BMI) (*p* = 0.02); and stunted children exhibited improved school attendance (*p* = 0.04). No treatment‐related effects were detected for height‐for‐age or other subtests of the Wide Range Achievement Test (WRAT)
60	(Lobato et al. 2012)	longitudinal, experimental study	6–10	Learning, memory, verbal fluency, and nonverbal intelligence	CPM, DAP‐III, WISC‐III	Hookworm	Brazil	2	Participants who received a targeted health education intervention demonstrated significantly higher scores on a structured knowledge questionnaire compared with controls (*p* < 0.05). However, no significant between‐group differences were observed on standardized cognitive assessments, including the Raven's Progressive Matrices, the Wechsler Intelligence Scale for Children (WISC‐III), and the Draw‐A‐Person Test (DAP‐III). These findings suggest that while the educational intervention enhanced conceptual understanding, it did not translate into measurable improvements in formal cognitive test performance.
61	(Thériault et al. 2014)	Cluster‐randomized controlled trial	Grade 5 students	Absenteeism	Teachers’ attendance logs	STH	Peru	9	In a controlled trial involving 1088 Peruvian schoolchildren, a health hygiene education intervention did not result in a reduction in school absenteeism over a 4‐month follow‐up period. Children with moderate‐to‐heavy Ascaris lumbricoides infection missed 2.4% more school days than their uninfected peers, while those with light hookworm infection missed 4.6% more days. Infections with *T. trichiura* and cases of polyparasitism were not significantly associated with absenteeism. Absenteeism increased with age, rising by 1.7% per additional year, and was highest among children in the lowest socioeconomic status (SES) quartile
62	(Berhe et al. 2009)	Cross‐sectional study	Mean age: 13.4 years	attentiveness	Structured questionnaire	*S. mansoni*	Ethiopia	7	High‐intensity *S. mansoni* infection, defined as >100 eggs per gram (epg), was significantly associated with severe abdominal cramps that impaired classroom attentiveness (OR = 3.3). Among infected children, 17.7% reported such symptoms, with listlessness and bloody‐mucoid stools frequently observed. Although malnutrition was more prevalent in endemic regions, it was not significantly associated with attentiveness. No significant associations were found between attentiveness and sex, age, co‐infection with soil‐transmitted helminths (STH), or hepatosplenomegaly
63	(Nokes et al. 1992)	Randomized, double‐blind, placebo‐controlled trial	9–12	attention, memory, processing, school attendance,	WISC, Fluency Test, MFFT, Listening Comprehension, Raven's Matrices	*T. trichiura*, *A. lumbricoides*, and *N. americanus*	Jamaica	9	In a randomized controlled trial involving 159 Jamaican children with moderate to heavy *T. trichiura* infection, anthelmintic treatment led to significant improvements in short‐term memory, assessed via Digit Span Forwards and Backwards, and long‐term memory retrieval, measured through verbal fluency tasks. No treatment effects were observed for arithmetic, coding, or comprehension subtests. Following intervention, treated children performed comparably to uninfected controls across memory domains. Cognitive improvements were independent of socioeconomic status, nutritional indicators, and baseline infection intensity.
64	(Al Serouri et al. 2000)	Randomized controlled trial/ natural trial	9–14	Speed of processing, attention, and working memory, School Achievement, school records, Absenteeism	Stroop test, visual search task, peg board task, verbal fluency task, speed of processing task, auditory working memory task, and visual memory task, WRAT	*P. falciparum*	Yemen	9	Among Yemeni schoolboys, asymptomatic Plasmodium parasitaemia was associated with reduced fine motor performance. While short‐term parasite clearance did not yield immediate cognitive improvements, children with high baseline parasite density demonstrated gains in memory and motor function following treatment. The findings suggest that longer‐term neurocognitive benefits may be plausible and warrant further investigation
65	(Rasoamanamihaja et al. 2016)	Cross‐sectional survey	7–10	School attendance	Head Teacher Report	*S haematobium, S. masssnsoni*	Madagascar	7	In a baseline survey of 1958 Malagasy children, *S. haematobium* infection was highly prevalent, affecting 30.5% of the cohort. While overall schistosomiasis prevalence did not differ significantly by school attendance status, heavy‐intensity *S. mansoni* infection was markedly more common among nonattenders (adjusted OR = 7.5; *p* = 0.037). No significant associations were observed between water, sanitation, and hygiene (WASH) indicators and infection status. The authors underscore the potential risk of excluding high‐burden children from school‐based mass drug administration (MDA) programs
66	(Nga et al. 2011)	A randomized, double‐blind, placebo‐controlled 2×2 factorial trial	6–8	working memory, attention, speed of information processing, executive function	WISC III, Raven's Colored Progressive Matrices test	*A. lumbricoides*, *T. trichiura*, Hookworm	Vietnam	9	In a randomized controlled trial involving 510 Vietnamese children, consumption of micronutrient‐fortified biscuits led to modest improvements in cognitive performance, with gains observed in Raven's Progressive Matrices (+0.86) and Digit Span Forward (+0.34). Cognitive benefits were more pronounced among anemic children, who demonstrated a greater increase in Raven's scores (+1.86). No significant effects were detected on other cognitive assessments or academic grades. Albendazole monotherapy did not yield cognitive improvements. However, the combined intervention of fortification and deworming significantly reduced the prevalence of Ascaris lumbricoides and *T. trichiura* compared with either strategy alone. Mid‐upper arm circumference (MUAC) increased slightly, though no significant changes were observed in height or weight
67	(Jardim‐Botelho et al. 2008)	Cross‐sectional study	6–11	Fluid intelligence, short‐term auditory attention, learning, and memory	Raven test, WISC‐III,	Hookworm, *A. lumbricoides, and* *S. mansoni*	Brazil	7	Moderate‐to‐high intensity *Ancylostoma duodena*le (hookworm) infection was significantly associated with reduced performance on the WISC‐III Coding subtest (OR = 3.20), with low‐intensity hookworm infection also demonstrating a strong association (OR = 3.71). Moderate‐to‐high intensity Ascaris lumbricoides infection was linked to poorer outcomes on Raven's Progressive Matrices (OR = 2.03), suggesting an impact on general intelligence. Children co‐infected with *A. lumbricoides* and hookworm exhibited lower scores on both the Arithmetic and Coding tasks compared with those with single‐species infections. The most pronounced cognitive impairment was observed in children with concurrent moderate *A. lumbricoides* and light hookworm infections, who showed markedly increased odds of poor Arithmetic performance (OR = 6.67). Haemoglobin levels did not confound these associations
68	(Ebenezer et al. 2013)	Cluster‐randomized, placebo‐controlled trial	Grade 4 school children	Attention	The code transmission test	*A. lumbricoides*, hookworm, and *T. trichiura*	Sri Lanka	9	In a cluster‐randomized controlled trial involving 1,190 Grade 4 children, a 6‐month school‐based intervention comprising deworming and iron supplementation significantly reduced the prevalence of Ascaris lumbricoides and *T. trichiura*. However, no improvements were observed in haemoglobin concentrations, anaemia prevalence, or performance on cognitive (code transmission) and educational assessments (mathematics and Tamil language). Notably, *A. duodenale* (hookworm) prevalence increased following treatment. Subgroup analyses revealed no significant intervention effects among children with high baseline worm burden or poor nutritional status
69	(Meremikwu et al. 2000)	Interventional (pre–post study)	8–10	Educational achievement, attendance rate,	Teacher‐Made Test, Attendance register	*S. haematobium*	Nigeria	7	In a 3‐year longitudinal cohort study involving 210 Nigerian children, repeated administration of praziquantel led to a marked reduction in *S. haematobium* prevalence (from 69.1% to 21.6%) and associated egg counts. Educational pass rates showed a statistically significant improvement (from 81.4% to 84.2%; *p* = 0.027), whereas school attendance declined marginally and without statistical significance (from 86.7% to 81.1%). The study authors advocate for routine praziquantel administration in endemic settings to enhance educational outcomes
70	(Ofosu and Ako‐nnubeng 2014)	Cross‐sectional	School‐aged children	School attendance	School registers	*A. lumbricoides*, *T. trichiura*, *A. duodenale*	Ghana	5	In a cohort of 286 Ghanaian schoolchildren, the prevalence of soil‐transmitted helminth (STH) infection was 6%. Following deworming, school absenteeism decreased by 8 percentage points, from 23.7% to 15.7%. Notably, all children with confirmed STH infection had missed school, underscoring the adverse effect of helminthiasis on attendance. The authors recommend complementary hygiene education and improved sanitation measures to sustain the observed reductions in absenteeism.
71	(Shang and Tang 2010)	Case‐control	9–12	Verbal comprehension, working memory, processing speed, and full‐scale IQ	WISC‐IV	Hookworm, *A. lumbricoides*, or *T. trichiura*	China	6	In a study of 1,031 Chinese schoolchildren, moderate‐to‐heavy soil‐transmitted helminth (STH) infections were significantly associated with deficits in verbal comprehension, working memory, processing speed, and full‐scale intelligence quotient (IQ). *A. duodenale* (hookworm) exerted the greatest impact on working memory, while *T. trichiura* infection was primarily linked to reduced processing speed. Despite these domain‐specific impairments, overall IQ scores remained within the borderline‐normal range
72	(Nampijja et al. 2020)	Cluster‐randomised controlled trial	5–9	Executive functions. (attention control, cognitive flexibility, working memory, inhibitory control, and planning)	Counting Span, Picture Search, Card Sort Test, Digit Span Backwards, Tower of London, and Delayed Inhibition Task	*S. mansoni*, *T. trichiura*, Hookworm, *A. lumbricoides*	Lake Victoria, Uganda	9	In a 2‐year randomized trial involving 384 Ugandan children, intensive deworming led to a reduction in *S. mansoni* prevalence; however, no significant improvements were observed in anthropometric measures or cognitive performance. Educationally relevant cognitive scores did not differ significantly between intervention groups, suggesting that reinfection and persistent environmental exposures may have attenuated potential benefits
73	(Mendy et al. 2015)	Cross‐sectional	12–16	Math, reading, visuospatial reasoning, verbal memory, working memory	WRATR, WISC‐R	*Toxoplasma gondii*	USA	7	Among 1755 children in a US cohort, seropositivity for *T. gondii* was associated with lower performance in reading and memory tasks. The adverse effect on memory was more pronounced in children with reduced serum vitamin E levels, indicating a potential modifying role of antioxidant status. No significant associations were observed with mathematical ability or visuospatial reasoning
74	(Singhi et al. 2018)	Prospective observational study	3–14	Cognitive abilities, Scholastic performance, intelligence	Parents’ interviews, teachers’ reports, grades obtained in school, comparison with immediate siblings, Goodenough‐Harris Draw‐a‐Man test	*Taenia solium*	India	9	In a cohort study of 500 Indian children diagnosed with neurocysticercosis, 22.2% exhibited below‐average academic performance, and 18.2% had intelligence quotient (IQ) scores below 70. Adverse cognitive and educational outcomes were significantly associated with the presence of multiple cerebral lesions, lesion calcification, recurrent seizures, and lower socioeconomic status. Notably, cognitive impairments persisted even after radiological resolution of lesions, indicating long‐term neurodevelopmental impact
75	(Sakti et al. 1999)	Cross‐sectional study	8–13	Cognitive abilities (working memory, sustained attention), motor function	Picture search, Number choice, Stroop, Categorical fluency, Digit‐span forwards, Digit‐span backwards, Corsi block, Free recall, Verbal analogies, Pegboard, Bead threading, Mazes	*A. lumbricoides, T. trichiura*, Hookworm	Indonesia	7	Among 432 Indonesian children, hookworm infection was significantly correlated with reduced performance across six cognitive assessments, notably including Fluency, Digit Span Forwards, Stroop Colour Word, and Mazes. The most pronounced effects were observed in older children and were concentrated within working memory domains. No significant associations were found with motor function or other cognitive measures. These cognitive deficits remained statistically robust after controlling for nutritional status and socioeconomic factors
76	(Mekheimar and Talaat 2005)	Case‐control	6–12	Attendance	% enrolled in school	*S. mansoni*	Egypt	5	In rural Egypt, children not enrolled in school exhibited lower haemoglobin levels, increased clinical indicators of vitamin deficiency, and a higher prevalence of schistosomiasis compared with their enrolled counterparts. School‐enrolled children demonstrated superior knowledge and attitudes regarding schistosomiasis; however, preventive practices and exposure levels remained suboptimal across both groups. Maternal education, alongside cultural and financial constraints, emerged as the primary determinant influencing school enrolment
77	(Hadidjaja et al. 1996)	Cluster‐randomized, placebo‐controlled trial	6–8	Short‐term memory, attention, and concentration. Learning	Ravens Colored Progressive Matrices, Wechsler Intelligence Scale for Children	*A. lumbricoides*	Indonesia	4	In slum communities of Jakarta, treatment with mebendazole for light‐intensity Ascaris infection resulted in significant improvements in visual reasoning and perceptual speed, as measured by PM‐C and Coding tasks. No cognitive enhancement was observed following health education alone or in combination with pharmacological intervention. Cognitive gains were attributable to reductions in helminth burden and remained independent of socioeconomic status and nutritional indicators
78	(Ahmed et al. 2012)	Pre–post intervention	6–13	School attendance/absenteeism	School attendance registers	*A. lumbricoides*, *T. trichiura*, Hookworm	Malaysia	9	In a cohort of 289 Aboriginal children, moderate‐to‐heavy soil‐transmitted helminth (STH) infections, primarily Ascaris and Trichuris, were significantly associated with elevated school absenteeism. Following albendazole administration, absenteeism rates declined by approximately 16%. Anaemia and limited paternal education were also identified as significant predictors of reduced school attendance. The study advocates for school‐based deworming initiatives as a strategic approach to enhance educational participation
79	(Terer et al. 2013)	Cross‐sectional	5–18	Achievement	Score in school functioning	*S. haematobium*	Kenya	7	In a study of 802 children residing in coastal Kenya, health‐related quality of life (HrQoL) scores were 2%–4% lower in villages with high *S. haematobium* prevalence. Within moderate‐prevalence settings, infection was significantly associated with reduced psychosocial HrQoL. Socioeconomic status and stunting emerged as key effect modifiers. The Pediatric Quality of Life Inventory Short Form 15 (PedsQL SF15) was found to be both reliable and operationally feasible for assessing the burden of neglected tropical diseases (NTDs) in this context
80	(Hutchinson et al. 1997)	Cross‐sectional study	9–13	School achievement	Wide Range Achievement Test	*T. trichiura*, *A. lumbricoides*	Jamaica	5	In a cohort of 800 Jamaican schoolchildren, infections with *T. trichiura* and Ascaris lumbricoides were significantly associated with reduced academic achievement. Anaemia and *A. lumbricoides* infection emerged as predictors of increased school absenteeism, while height‐for‐age was positively correlated with arithmetic performance. The authors advocate for integrated health and nutrition interventions as a strategy to enhance educational outcomes

Abbreviations: BASC, Behavior Assessment System for Children; CB, Corsi Block; CBCL, The Child Behavior Checklist; CFT, Category Fluency Test; CNT, contingency naming test; CRT, choice reaction time; CTMT, Comprehensive Trail‐Making Test; DAP, Draw‐A‐Person Test; DAP‐III, the Draw‐a‐Person Test third edition; DCCS, Dimensional Change Card Sort Test; DS, Digit Span; DSF, Digit Span Forwards; EGMA, the Early Grade Maths Assessments; EGRA, the Early Grade Reading Assessment; Flanker, Flanker Test; FR, free recall; IQ, intelligence quotient; KABC‐II, Kaufman Assessment Battery for Children Second Edition; LS, List Sorting; MC‐HOME, middle childhood home observation for the measurement of the environment; MFFT, Matching Familiar Figures Test; N, number; PBT, pegboard task (dominant and nondominant hand); PC, Pattern Comparison; PMA, Primary Mental Abilities; PNIT, Philippines Non‐Verbal Intelligence Test; PPVT, Peabody Picture Vocabulary Test; PSM, Picture Sequence; R, reasoning; RCPM, Raven's Colored Progressive Matrices; S, space; S/N, serial; NOS, Newcastle–Ottawa Scale; SCWT, Stroop Color and Word Test; SOPT, self‐ordered pointing test; SS = silly sentences; STH, Soil‐transmitted helminth; SWLT, Spanish word learning task; TEA‐Ch, Tests of Everyday Attention for Children; TIMSS, Trends in International Mathematics and Science Study; TOVA, test of variables of attention; TPT, Tactual Performance Test; TRS, Teacher Rating Scales; V, verbal meaning; W, word fluency; WF, word fluency; WISC III, Wechsler Intelligence Scale for Children; WISC‐III, Wechsler Intelligence Scale, third edition for children; WISCIV, Wechsler Intelligence Scale for Children—Fourth Edition; WISC‐R, Wechsler Intelligence Scale for Children‐Revised; WRAML, Wide Range Assessment of Memory and Learning; WRAT, Wide Range Achievement Test; WRAT‐3, Wide range achievement test‐third edition; WRAT‐R, Wide Range Achievement Test‐Revised.

### 2.4 Quality Assessment and Risk of Bias

To ensure a rigorous and standardized evaluation of study quality and bias, the Newcastle–Ottawa Scale (NOS) and the Cochrane Risk of Bias Tool were employed to appraise methodological rigor, assess potential sources of bias, and maintain consistency across observational and interventional studies included in the review. The NOS provides design‐specific criteria to assess the quality of observational studies, including cohort, case‐control, and cross‐sectional designs.

Each paper was independently evaluated by two reviewers based on three major criteria: (i) selection of study groups, (ii) comparability of cohorts or cases/controls, and (iii) ascertainment of exposure or outcome. A maximum of nine stars was allocated per study. For case‐control and cohort studies, one star was awarded for each numbered item within the selection and exposure/outcome categories. The comparability section was modified to account for the confounding effects of age, sex, nutrition, and socioeconomic status on the relationship between parasitic infection and cognitive or educational outcomes. Comparability for these factors was achieved either through study design (e.g., age or sex restrictions, randomization in RCTs) or analytical methods such as stratified analyses or multivariable adjustments in regression models, with a maximum of two stars awarded.

For cross‐sectional studies, a modified NOS was applied, awarding up to three stars for selection, two stars for comparability, and two stars for outcome assessment. Each study's raw quality score was rescaled to a 9‐point scale, following established precedents (Lo et al. 2014).

Cohort and case‐control studies were evaluated using eight sub‐criteria, with quality ratings categorized as follows: high quality (7–9 stars), moderate quality (5–6 stars), and low quality (fewer than 5 stars). For cross‐sectional studies, a modified assessment scale was used to account for their distinct methodological characteristics. This adapted version typically assessed domains such as sample representativeness, measurement reliability, and adjustment for confounding variables. Quality was similarly classified as high (7–9 stars on a 9‐point scale), moderate (5–6 stars), or low (fewer than 5 stars). The methodological quality of the included studies varied considerably. Most demonstrated moderate rigor (scores 5–6), with adequate participant selection and reasonable comparability achieved through adjustment for age, sex, nutrition, or socioeconomic status, though broader control for contextual determinants such as school infrastructure or household environment was often lacking. A smaller subset attained high quality (scores ≥7), distinguished by robust outcome measurement, standardized cognitive assessment protocols, and comprehensive reporting of educational performance. In contrast, lower‐quality studies (scores <5) were limited by small sample sizes, incomplete reporting of cognitive outcomes, or insufficient adjustment for confounders, and were therefore excluded from the final synthesis.

### 2.5 Ethical Considerations and Data Synthesis

Two reviewers independently carried out the quality appraisal. Based on their assessments, studies were either included in or excluded from the final review. Any disagreements regarding study eligibility were resolved through discussion, with the involvement of a third reviewer when necessary. Due to anticipated heterogeneity in clinical, methodological, and statistical aspects, a narrative synthesis approach was adopted, as the diversity in participant characteristics, interventions, outcomes, study designs, and risk of bias rendered a quantitative synthesis inappropriate. Ethical approval was not required for this review, as it relied solely on data from publicly accessible, published sources.

## 3 RESULTS

A comprehensive synthesis of 80 studies demonstrates consistent associations between parasitic infections and impairments across multiple domains of cognition. Memory deficits were reported in 67% of studies, with younger children aged 5–9 years emerging as the most prevalent group, where heavy *Schistosoma* and *Trichuris* infections were linked to poorer recall and fluency; intervention trials, however, documented improvements in working memory following treatment (Table 2; Table 4). Attention was the most consistently affected domain, with about 75% of studies identifying impairments. Children with malaria and helminth infections exhibited slower reaction times and reduced vigilance on Stroop and Flanker tasks, often compounded by anemia and stunting (Table 3; Table 4). Executive function deficits were observed in approximately 67% of studies, manifesting as difficulties in planning, inhibitory control, and cognitive flexibility. These impairments were most pronounced in cases of polyparasitism and high infection intensity, while intervention outcomes were mixed, showing gains in dynamic tasks but limited improvements in static measures (Table 3; Table 4). IQ and academic achievement were reduced in about 70% of studies, most frequently in children with STH and schistosomiasis, as assessed by standardized tools such as the Wechsler Intelligence Scale for Children—Fourth Edition (WISC‐IV) and the Wide Range Achievement Test (WRAT). School records consistently revealed higher absenteeism among infected children, and even light infections were sometimes associated with unexpectedly poor outcomes, suggesting that low parasite burdens can still disrupt learning (Table 2; Table 3). Reaction time and motor coordination showed the strongest consistency, with 80% of studies reporting impairments. Children with malaria and helminth infections demonstrated slower reaction times and impaired motor coordination, which often improved following treatment, though reinfection limited sustained benefits (Table 4). Regionally, sub‐Saharan Africa contributed more than 70% of the evidence base, with similar trends observed in Asia and Latin America. At the same time, additional findings from the USA highlighted the impact of *Toxoplasma gondii* on reading and memory performance (Table 5).

**TABLE 2:** Distribution of study characteristics, cognitive domains, and associations by age group.

Age group	No. of studies	Positive association	Null association	Cognitive domains assessed	Cognitive assessment tools used
**5–9 years**	18	13 (72%)	5 (28%)	Memory, attention, reaction time	Raven's Progressive Matrices, Digit Span, Fluency, Stroop
**10–14 years**	38	28 (74%)	10 (26%)	IQ, executive function, school performance	Wechsler Intelligence Scale for Children (WISC), Stroop Test, WRAT
15–18 years	10	5 (50%)	5 (50%)	Motor coordination, academic achievement	Trail Making Test, academic records, PedsQL
Mixed ranges (5–16, 7–19, 5–18)	14	10 (71%)	4 (29%)	Academic performance, memory, executive function	WRAML, PNIT, school records, teacher reports

Abbreviations: IQ, Intelligence Quotient; PedsQL, Paediatrics Quality of Life; PNIT, Philippines Non‐Verbal Intelligence Test; WRAML, Wide Range Assessment of Memory and Learning; WRAT, Wide Range Achievement Test.

**TABLE 3:** Cognitive and academic outcomes by parasite type.

Parasite type	No. of studies	Positive association	Null association	Cognitive domains affected	Biomarkers reported
*Schistosoma* spp.	29	20 (69%)	9 (31%)	Memory, attention, executive function, school attendance	Hemoglobin, ferritin, infection intensity (egg counts)
Soil‑transmitted helminths (STHs)	26	18 (69%)	8 (31%)	IQ, academic performance, attention, processing speed	Hemoglobin, anthropometry, stool microscopy
*Plasmodium* spp.	15	11 (73%)	4 (27%)	Reaction time, motor coordination, memory, executive function	Parasite density, cerebral malaria diagnosis, TNF levels
Other parasites	10	8 (80%)	2 (20%)	Language, motor skills, psychosocial outcomes	Nutritional markers, stool antigen tests, vitamin E levels

Abbreviation: IQ, intelligence quotient.

**TABLE 4:** Cognitive domains affected by parasite infection.

Cognitive domain	No. of studies	Positive association	Null association	Assessment tools
Memory	28	20 (71%)	8 (29%)	Digit Span, Word Recall, WRAML, Verbal Fluency
Attention	32	24 (75%)	8 (25%)	Stroop Test, Continuous Performance Test, Flanker, TEA‑Ch
Executive function	22	15 (68%)	7 (32%)	Wisconsin Card Sorting Test, Tower of London, PNIT
IQ scores	26	18 (69%)	8 (31%)	WISC, Raven's Matrices, Stanford‑Binet
Reaction time and motor coordination	15	12 (80%)	3 (20%)	Trail Making Test, Finger Tapping, Mazes

Abbreviations: PNIT, Philippines Non‐Verbal Intelligence Test; TEA‐Ch, Tests of Everyday Attention for Children; WISC, Wechsler Intelligence Scale for Children; WRAML, Wide Range Assessment of Memory and Learning.

**TABLE 5:** Region‑specific findings (sub‑Saharan Africa vs. other regions).

Region	No. of studies	Positive association	Null association	Dominant parasites	Cognitive/Academic domains affected	Key contextual modifiers
Sub‐Saharan Africa (East, West, Southern, North Africa combined)	57	41 (72%)	16 (28%)	*Schistosoma* spp., *Plasmodium* spp., STHs, *Tunga penetrans*	Memory, attention, IQ, executive function, school performance, absenteeism	Reinfection risk, anemia, stunting, SES, hygiene practices, maternal education
Asia (China, Philippines, Indonesia, Vietnam, Cambodia, India, Malaysia, Yemen)	17	12 (71%)	5 (29%)	*A. lumbricoides*, *T. trichuria, Hookworm*, *S. japonicum*, *T. solium*	Working memory, processing speed, IQ, school performance	Nutritional status, micronutrient fortification, lesion burden (neurocysticercosis), SES
Latin America and the Caribbean (Brazil, Jamaica, Peru, Saint Lucia)	6	4 (67%)	2 (33%)	*P. vivax, Hookworm*, *T. trichuria*, *S. mansoni*	IQ, memory, school performance, absenteeism	Anemia, recurrent malaria episodes, school quality, language factors
USA	1	1 (100%)	0	*T. gondii*	Reading, memory	Antioxidant status (vitamin E), SES

Abbreviations: IQ, Intelligence Quotient; SES, socioeconomic status.

Overall, the review highlights the substantial, yet frequently overlooked, burden of neglected tropical parasitic diseases (NTDs), which adversely affect cognitive development, limit educational attainment, and hinder long‐term socioeconomic prospects for affected children. The results consistently demonstrate that parasitic infections are significantly associated with impairments in neurocognitive function and academic performance among school‐aged children in endemic regions

### 3.1 Age‐related Differences

Children aged 6–9 years frequently demonstrated impairments in memory and attention when infected with helminths or malaria, as evidenced by performance on standardized assessments such as the Digit Span and Raven's Progressive Matrices (Nokes et al. 1999; Jukes et al. 2002; Kariuki et al. 2014). These findings are consistent with developmental neuroscience perspectives that emphasize early childhood as a critical stage for the consolidation of working memory and the development of attentional control (Buss et al. 2018). Nevertheless, some studies reported no significant associations, particularly when infection intensity was relatively low or when nutritional confounders were adequately accounted for (Tandoh et al. 2020; Hürlimann et al. 2014). Notably, these studies were rated as high quality on the Newcastle–Ottawa scale (scores ≥7), indicating that the absence of associations cannot be attributed to methodological weakness alone. Rather, such findings may reflect genuine variability in infection intensity, nutritional status, or the presence of contextual resilience factors.

For children between 10 and 14 years, evaluations using the WISC, Behavior Assessment System for Children (BASC‐2), and the Stroop Test revealed notable deficits in intelligence quotient (IQ), executive functioning, learning difficulties, attention problems, and school‐related behavioral deficits (Jukes et al. 2002; Musuva et al. 2017). Comparable evidence from Asia supports these findings: for example, helminth infections among Filipino children were independently associated with poorer cognitive performance, including deficits in learning and memory, even after adjusting for nutritional and socioeconomic confounders (Ezeamama et al. 2005).

Among adolescents aged 15 to 18 years, findings were heterogeneous. In Filipino cohorts, baseline polyparasitism was linked to lower performance on the Philippines Non‐Verbal Intelligence Test (PNIT). Complementary assessments using the Wide Range Assessment of Memory and Learning (WRAML) further demonstrated impairments in verbal memory and fluency, highlighting the broad cognitive impact of multiple concurrent infections (Ezeamama et al. 2012). In contrast, Kenyan studies involving older adolescents yielded mixed findings. While *S. haematobium* infection was linked to reduced psychosocial quality of life, its association with academic achievement was inconsistent, indicating that contextual factors such as stunting, anemia, and socioeconomic disadvantage may influence the relationship between infection and educational outcomes (Terer et al. 2013). This inconsistency may be attributable to compensatory influences such as educational opportunities or resilience factors, highlighting the need for longitudinal research to better capture potential delayed or cumulative effects.

### 3.2 *Schistosoma* Species and Their Influence on Cognition

Robust evidence demonstrates that infections with *S. haematobium*, *S. mansoni*, and *S. japonicum* are associated with deficits in memory, attention, executive function, and academic performance. Studies from Tanzania and Nigeria reported poorer outcomes on Digit Span tasks assessing short‐term memory and slower responses on reaction time tasks (Jukes et al. 2002; Inyang‐Etoh et al. 2018). Egyptian and Kenyan cohorts also documented reductions in IQ, reasoning, academic difficulties, attention problems, and learning problems, as measured by the WISC and BASC, alongside increased absenteeism in school records (Nazel et al. 1999; King et al. 2020). Consistent results have also been reported in southern Africa, where studies from Zimbabwe (Loveridge et al. 1948) and South Africa (Castle et al. 1974) linked *Schistosoma* infection to diminished educational achievement and impaired cognitive functions, including executive processing, learning, and memory retention, using School test scores, WRAML, and the PNIT.

Situating these findings within a broader context, comparative findings from Asia and Latin America enrich interpretation. Evidence from Asia further confirmed that *S. japonicum* infection in China negatively affected reasoning and learning capacity, particularly among stunted and anemic children assessed by the WISC‐IV (Nokes et al. 1999). Studies in the Philippines documented impairments in learning, memory, and verbal fluency among infected children evaluated by WRAML, PNIT, and verbal fluency task (VFT) (Ezeamama et al. 2012; Ezeamama et al. 2005), while findings from Saint Lucia revealed that children affected by schistosomiasis, particularly those with multiple parasitic co‐infections, showed increased school absenteeism and diminished classroom performance on the Nelson Reading Skills Test (NRST), underscoring the direct influence of infection burden and attendance on academic outcomes (Epstein and Weisbrod 1974).

However, several studies have questioned the generalizability of these deficits. In Nigeria, Ejezie and Ade‐Serrano (1981) and Ekanem et al. (1994) found no significant associations between *S. haematobium* infection and school attendance, academic performance, measured using Average grades scored, school attendance records, and teacher‐given tests. Both studies were rated as moderate quality (NOS 5–6), attributing null findings to low infection burden and possible regional variation in parasite strains, low transmission intensity, and partial immunity acquired through prior exposure.

The cognitive decline associated with Schistosoma infections arises from several factors related to the chronic nature of the disease. For example, *S. haematobium* infection can result in anemia, particularly iron‐deficiency anemia, due to sustained blood loss from the urinary tract (hematuria) (Butler et al. 2012). While intestinal schistosomiasis caused by *S. mansoni* and *S. japonicum* can lead to gastrointestinal bleeding and nutrient malabsorption, exacerbating iron deficiency and overall malnutrition (Ghazy et al. 2025). Since iron plays a vital role in neurotransmitter production and myelin synthesis, its deficiency negatively affects brain function, impairing cognitive skills such as attention, memory, and processing speed (Pivina et al. 2019). Chronic infection also provokes persistent systemic inflammation, with elevated cytokines such as TNF‐α, IL‐6, and IFN‐γ driving neuroinflammation and disrupting neuronal signaling, as the immune system continuously responds to circulating parasite antigens and trapped eggs (Kasambala et al. 2023). This inflammatory state can interfere with nutrient absorption, contributing to malnutrition and potentially causing neuroinflammation, which further compromises cognitive processing (Gasparotto et al. 2021). The associated symptoms of schistosomiasis, including chronic fatigue, abdominal discomfort, hematuria, and general malaise, can significantly lower a child's energy levels, reducing their ability to concentrate and actively engage in classroom activities (Jukes et al. 2002). As a result, schistosomiasis contributes to poor school attendance and declining academic performance (Partnership for Child Development 2002).

### 3.3 Soil‐Transmitted Helminths and Cognitive Outcomes among School Children

The result of this review revealed that STH infections, including *Ascaris lumbricoides*, hookworm *(Ancylostoma duodenale, Necator americanus), Trichuris trichiura*, and *Strongyloides stercoralis*, have been widely associated with reductions in IQ, memory, attention, and academic achievement. Evidence from Ghana (Tandoh et al. 2020; Ofosu and Ako‐nnubeng 2014), Côte d'Ivoire (Hürlimann et al. 2014), Ethiopia (Alemayehu et al. 2025; Melesse et al. 2017; Degarege et al. 2022), and South Africa (Gall et al. 2017; Beckmann et al. 2021; Gerber et al. 2021) consistently report deficits in attention, memory, executive function, and processing speed. These impairments have been evidenced by reduced IQ scores measured with the WISC and RPM, alongside academic difficulties and increased absenteeism documented in school records. A study conducted in Jamaica (Hutchinson et al. 1997) also reveals that concurrent infections with *T. trichiura* and *A. lumbricoides* markedly hindered academic performance, as measured by school records and teacher reports.

Although Africa carries the greatest burden of STH infections, comparative evidence from Asia and Latin America provides valuable contextual insights. In Indonesia and Malaysia, studies reported reductions in working memory and increased school absenteeism among children infected with *A*. *lumbricoides*, *T. trichiura*, and hookworm (Sakti et al. 1999; Hadidjaja et al. 1996; Ahmed et al. 2012). In China, moderate‑to‑heavy STH infections were significantly associated with deficits in verbal comprehension, working memory, and processing speed (Shang and Tang 2010). Evidence from Cambodia and Vietnam further demonstrated that infection intensity and nutritional status shaped cognitive outcomes, with impairments observed in reasoning and working memory tasks (Kuong et al. 2016; Nga et al. 2011). Similarly, research from the Philippines showed that helminth infections were independently linked to poorer learning and memory performance, even after controlling for nutritional and socioeconomic confounders (Ezeamama et al. 2005).

Despite evidence of helminth‐related cognitive impairment in some settings, multiple studies have reported null or inconsistent associations. Jumbe and Siwila (2024) found no significant relationship between light‐intensity STH infections and working memory, processing speed, or academic achievement among Zambian children, with outcomes measured by records of school scores obtained by children. This study was rated as moderate quality on the Newcastle–Ottawa scale (score 5), reflecting limitations in sample size and inadequate adjustment for confounding variables. These methodological weaknesses may have contributed to the absence of significant associations, and the authors themselves highlight the need for larger, more rigorously designed investigations to more accurately evaluate potential cognitive and educational effects.

Similarly, Opeyemi Olopade et al. (2018) observed no differences in IQ scores between infected and uninfected Nigerian pupils. This study was rated as high quality on the Newcastle–Ottawa Scale (score 7), indicating that the null findings are methodologically robust. The authors suggested that the absence of measurable effects may be attributable to low infection intensity and potential protective influences of hygiene practices, noting that regular handwashing was significantly protective. Findings from intervention trials have also been mixed. In Guatemala and Jamaica, Watkins et al. (1996) and Gardner et al. (1996) documented no significant cognitive improvements following deworming, despite effective parasite clearance, with outcomes assessed using the Peabody picture vocabulary test (PPVT), WISC subtests, and School attendance records. This study was rated as high quality on the Newcastle–Ottawa scale (score 9), indicating that the null findings are methodologically robust. The authors proposed that the neurocognitive impact of *Trichuris* infection may be minimal in well‑nourished pediatric populations, thereby emphasizing the moderating role of nutritional context in shaping infection‑related outcomes.

Mechanistically, STH infections impair cognitive development primarily through nutritional deficiencies and chronic inflammation (Ezeamama et al. 2018). Hookworm infection contributes to iron‐deficiency anemia via sustained intestinal blood loss, reducing oxygen delivery to the brain and impairing cognitive processes dependent on adequate iron levels (Usang et al. 2025; Leung et al. 2024). Infections with *A. lumbricoides* and *T. trichiura* further compromise nutrient absorption, exacerbating malnutrition and energy deficits essential for brain growth and function (Wardiyah et al. 2023). Similar to schistosomiasis, STH infections provoke persistent low‐grade inflammation, diverting metabolic resources away from neurodevelopmental processes and contributing to neuroinflammation (Piazzesi and Putignani 2023). Clinical manifestations such as lethargy, abdominal discomfort, diarrhea, and general ill‐health further diminish children's ability to concentrate, engage in classroom activities, and maintain regular attendance, ultimately impairing academic performance.

### 3.4 *Plasmodium falciparum*, Other Protozoa Infections, and Their Impact on Cognition Among School Children

This review found that infections caused by *Plasmodium* species, particularly *P. falciparum*, are consistently associated with impairments in working memory, executive function, attention, reaction time, IQ, and academic achievement.

Evidence from Uganda (Bangirana et al. 2011; Nankabirwa et al. 2013; Chandy et al. 2009) demonstrated persistent deficits in working memory measured by the Kaufman assessment battery for children second edition (KABC‐II), executive function assessed with the Test of Variables of Attention (TOVA), sustained attention, abstract reasoning captured by the code transmission test, and Ravens matrices, even years after cerebral malaria episodes. Kenyan and Ghanaian cohorts reported reduced IQ scores on the Self‐ordered pointing test (SOPT), declines in school performance in the cumulative end‐of‐term examination results, arithmetic test, and low attendance documented in school records (Kariuki et al. 2014; Donkoh et al. 2023; Orish et al. 2018).

Although Africa bears the greatest burden of malaria and protozoan infections, comparative evidence from Asia and Latin America provides valuable contextual insights. In Brazil, malaria was identified as an independent predictor of poor school performance, with recurrent episodes associated with reduced teacher‐evaluated scores (Vitor‐Silva et al. 2009). In Yemen, children with high baseline parasite density demonstrated poorer memory and motor function (Al Serouri et al. 2000). Evidence from India further highlighted long‑term impairments in problem‑solving and communication skills among children with protozoan co‑infections such as neurocysticercosis (Singhi et al. 2018). These findings highlight that the cognitive impact of malaria and protozoan infections is not confined to Africa but reflects broader regional variations in transmission intensity, co‑infection patterns, and educational environments. The neurological consequences of cerebral malaria are particularly severe, with long‐term impairments in planning, problem‐solving, and processing speed well‐documented (Chandy et al. 2009; Kariuki et al. 2014). Importantly, even asymptomatic malaria infections have been associated with subtle declines in working memory and sustained attention measured using the code transmission test, Ravens matrices, Dimensional Change Card Sort Test (DCCS), and Flanker Test (Nankabirwa et al. 2013; Johnson et al. 2024), highlighting that cognitive effects are not limited to severe cases.

Beyond malaria, protozoan infections such as *Giardia lamblia* and *Entamoeba histolytica* have also been linked to reduced IQ measured by the Stanford‐Binet intelligence scale, and poorer school performance and attendance recorded in grade scores and school attendance records (Morad and Allam 2018; Melesse et al. 2017). However, some studies reported null associations. For example, Halliday et al. (2012) found no significant relationship between *P. falciparum* infection and cognitive or educational outcomes in Kenyan children, measured on the Tests of Everyday Attention for Children (TEA‐Ch) and RPM task. This study was rated as high quality on the Newcastle–Ottawa scale (score 7), confirming the robustness of the null findings. The authors observed that educational performance was more strongly influenced by socioeconomic status, parental education, and classroom infrastructure, suggesting that these contextual determinants may exert a greater influence than the direct neurocognitive effects of malaria infection in this setting.

The cognitive effects of *P. falciparum* infection are largely attributable to its direct impact on neurological health. Cerebral malaria is characterized by parasitic sequestration in brain capillaries, triggering inflammation, cerebral edema, and localized hypoxia, all of which contribute to neuronal damage and long‐term cognitive deficits (Luzolo and Ngoyi 2019; Song et al. 2022). Furthermore, even uncomplicated malaria cases can induce severe anemia, reducing oxygen transport to the brain and impairing cognitive function (Rosa‐Gonçalves et al. 2022; Halliday et al. 2012). Systemic inflammation and febrile episodes further contribute to fatigue, decreased concentration, and poor classroom engagement (Reis and Castro‐Faria‐Neto 2022). Protozoan infections, such as giardiasis, primarily affect cognition through chronic malabsorption and nutritional deficiencies, indirectly impeding brain development (Kanokwanvimol 2017; Gutiérrez and Bartelt 2024). The recurrent nature of these infections exacerbates school absenteeism and disrupts learning continuity, ultimately leading to diminished academic outcomes. The cumulative effects of these infections highlight the need for comprehensive health interventions, including improved disease prevention strategies, nutritional support, and educational accommodations, to mitigate their impact on cognitive function and academic success in affected populations.

### 3.5 Other Parasitic Infections and Their Influence on Cognition among School Children

Besides schistosomiasis, soil‐transmitted helminths, and protozoan infections, other parasitic diseases are associated with cognitive and educational achievements among school‐aged children.

Tungiasis (*T. penetrans*) has been associated with increased absenteeism and lower academic performance, as evaluated by the class teacher's attendance register, and Exam scores recorded.

Evidence from Kenya, Uganda, and Rwanda documented increased absenteeism, reduced classroom participation, and lower academic performance, as measured through attendance registers, trimester average scores, and teacher‑recorded exam results (Nsanzimana et al. 2019; Elson et al. 2023). Beyond absenteeism, domain‑specific impairments have also been reported. In Kenya and Uganda, tungiasis was linked to deficits in literacy, language, cognitive flexibility, and working memory, assessed using standardized tools such as the Early Grade Reading Assessment (EGRA), Culture Fair Test (CFT), Comprehensive Trail Making Test (CTMT), Stroop Color Word Test (SCWT), backward digit span, bead threading, and the Child Behavior Checklist (CBCL) (Otieno et al. 2023; Elson et al. 2025). Physical morbidity, including pain, itching, and secondary infections, further constrained concentration and engagement, reinforcing the educational burden of this neglected parasitic disease.

*Enterobius vermicularis* (pinworm) infection has been linked to reduced scholastic achievement, as evaluated by the first‐term examination report, and higher rates of school absenteeism recorded in school registers. Studies from Egypt and Ethiopia consistently reported lower cognitive scores among infected children, although some findings became nonsignificant after adjustment for nutritional status (El‐Masry et al. 2007; Degarege et al. 2022). Comparative studies reinforce these findings. In India, neurocysticercosis was associated with impairments in cognitive abilities, scholastic performance, and IQ measured using parents’ interviews, teachers’ reports, grades obtained in school, comparison with immediate siblings, and the Goodenough‐Harris Draw‐a‐Man test, with deficits linked to cerebral lesions and recurrent seizures (Singhi et al. 2018). In the USA, *T. gondii* infection has been associated with poorer reading and memory tasks, measured using the Wide Range Achievement Test‐Revised (WRAT‐R) and the Wechsler Intelligence Scale for Children‐Revised (WISC‐R) (Mendy et al. 2015).

Protozoan and other parasitic infections impair cognition through both nutritional and neurological pathways. Chronic malabsorption associated with Giardiasis and amoebiasis leads to deficiencies in iron, folate, and vitamin B12, nutrients essential for neurotransmitter synthesis and myelin formation (Kanokwanvimol 2017; Gutiérrez and Bartelt 2024). Neurocysticercosis causes direct neurological damage through seizures, neuroinflammation, and structural brain lesions, resulting in long‐term deficits in memory, executive function, and communication skills (Singhi et al. 2018). In toxoplasmosis, immune‐mediated neuroinflammation and oxidative stress contribute to selective impairments in memory and reading ability (Mendy et al. 2015).

Clinical manifestations such as recurrent diarrhea, abdominal pain, seizures, and fatigue further reduce classroom engagement and attendance, compounding the educational impact. The physical morbidity of tungiasis, including severe itching, pain, inflammation, and secondary bacterial infections, hinders concentration, participation in school activities, and regular attendance (WHO 2022; Elson et al. 2024). In addition, psychological effects such as reduced self‐esteem and social stigma exacerbate barriers to learning and ultimately hinder academic progress and overall cognitive development (Alderton et al. 2024; Elson et al. 2025; Stephen 2023).

### 3.6 Intervention Outcomes and Their Heterogeneity

One of the most important findings from this review highlights the variability in cognitive and educational outcomes following intervention studies, reflecting differences in infection intensity, geographical context, intervention design, and the sensitivity of assessment instruments. Intervention studies targeting parasitic infections among school‐aged children have employed diverse methodological approaches, including randomized controlled trials (RCTs) (Nokes et al. 1999; Clarke et al. 2017), quasi‐experimental school‐based programs (Meremikwu et al. 2000; Ezeamama et al. 2012), and observational follow‐ups (de Clercq et al. 1998; Vitor‐silva et al. 2009), with outcomes differing across parasite groups.

In relation to schistosomiasis, most interventions implemented school‐based mass drug administration (MDA) with praziquantel, generally delivered as single‐dose (40 mg/kg) regimens (Nokes et al. 1999; Grigorenko et al. 2006; Musuva et al. 2017). RCTs conducted in Kenya and China demonstrated short‐term improvements in working memory and attention, assessed using the Fluency and Free Recall task, and the BASC, with follow‐up periods ranging from 6 months to 1 year (Nokes et al. 1999; Musuva et al. 2017). Similarly, Tanzania studies reported improvements or superior gains in dynamic cognitive functions, including syllogistic reasoning and sorting, relative to untreated peers, after 6–12 months of follow‐up, but no significant differences were observed between groups in academic achievement outcomes (Grigorenko et al. 2006). In the Philippines, school‑based administration of praziquantel (40 mg/kg, single dose) and albendazole (400 mg, single dose) was evaluated within a prospective cohort design, with follow‑up assessments of 6 and 12 months. Improvements were noted in verbal memory and fluency, as measured by the WRAML, indicating partial reversibility of infection‑related cognitive deficits (Ezeamama et al. 2012). By contrast, a 3‐year longitudinal study conducted in Nigeria reported modest or null effects; repeated administration of praziquantel was associated with a significant improvement in educational pass rates assessed by a teacher‐made test, but school attendance declined marginally in the attendance register, and this was not statistically significant (Meremikwu et al. 2000).

For STHs, interventions predominantly involved albendazole (400 mg single dose) or mebendazole, administered either as single doses or repeated cycles within school‐based deworming programs (Nokes et al. 1992; Watkins et al. 1996; Hadidjaja et al. 1996; Simeon et al. 1995). RCTs have yielded both positive and null findings regarding cognitive and academic outcomes. In Jamaica, albendazole treatment was associated with improvements in short‑ and long‑term memory tasks after 3 months of follow‑up (Nokes et al. 1992), while in Indonesia, albendazole produced gains in digit span and IQ among children with mild infections over the same period (Sari et al. 2012). Cluster‑RCTs in Indonesia further reported mebendazole‑related improvements in reasoning and perceptual speed at 6 months of follow‑up (Hadidjaja et al. 1996). In contrast, other trials documented limited or no benefits. In Guatemala, albendazole effectively eliminated *A. lumbricoides* but showed limited efficacy against *T. trichiura*, with no significant improvements in reading ability, receptive vocabulary, Peabody Picture Vocabulary Test (PPVT) scores, or school attendance after 6 months (Watkins et al. 1996). Similarly, in Jamaica, children with light to moderate *T. trichiura* infection exhibited only minimal impairment. Albendazole treatment did not produce measurable improvements in learning, academic achievement, or school attendance over 6 months, as assessed using the WRAT, digit span tasks, and school registers (Simeon et al. 1995; Gardner et al. 1996).

Malaria interventions included chemoprevention with artemisinin‐based combination therapies (ACTs) and older regimens such as chloroquine or sulfadoxine‐pyrimethamine (SP) or integrated parasite control programs delivered through school‐based or community platforms (Bangirana et al. 2011; Clarke et al. 2017; Orish et al. 2018; Chandy et al. 2009; Kariuki et al. 2014; Johnson et al. 2024). A cluster‑RCT conducted in Mali showed that integrated parasite control markedly reduced parasitaemia and anemia among school children, with measurable improvements in sustained attention as assessed through standardized tasks (TEA‐Ch) over a 12‐month follow‐up period (Clarke et al. 2017). In contrast, cohort and case‐control studies conducted in Uganda and Kenya reported only limited or domain‐specific cognitive improvements following malaria treatment. Children who experienced severe malaria with neurological involvement continued to show persistent impairments in executive functioning and attentional capacity despite therapeutic intervention, even at 3‐month follow‐up using the Stroop test and BASC (Bangirana et al. 2011; Kariuki et al. 2014) and 2‐year follow‐up using the KABC‑II, TOVA, and Tactual Performance Test (TPT) (Chandy et al. 2009). Likewise, in Ghana, asymptomatic *P. falciparum* infection was linked to reduced arithmetic performance, and antimalarial treatment did not fully restore these deficits, indicating that infection clearance alone may be insufficient to recover cognitive outcomes in settings characterized by anemia and educational disadvantage (Orish et al. 2018). More recent evidence implicates inflammatory pathways, with elevated TNF levels independently predicting lower performance on attention and working memory tasks, such as the Flanker, DCCS, and Picture Sequence Memory tests (Johnson et al. 2024).

For other parasitic infections, intervention designs were more limited. Tungiasis studies were primarily community‐based observational interventions, focusing on topical dimeticone or manual extraction, with outcomes measured in school attendance records over short follow‐up periods of weeks to months (Nsanzimana et al. 2019; Otieno et al. 2023). Neurocysticercosis interventions generally involved albendazole combined with corticosteroids in hospital‐based trials, which reduced lesion burden but left persistent deficits in executive function and language, assessed using the Goodenough‐Harris Draw‐a‐Man test, after follow‐up durations of 6–18 months (Singhi et al. 2018).

These findings demonstrate that intervention outcomes are influenced not only by parasite group and drug regimen but also by study design, follow‐up duration, and the choice of cognitive assessment tools. Short‐term RCTs frequently identified immediate improvements in attention and processing speed, whereas longer follow‐ups revealed persistent deficits in broader IQ measures. This heterogeneity underscores the need for standardized intervention designs incorporating extended follow‐up periods and sensitive cognitive instruments to ensure comparability across settings and more accurate evaluation of long‐term educational impacts.

### 3.7 Policy Implications

The variability in outcomes across intervention studies underscores the necessity of integrated policies that combine biomedical treatment with complementary public health measures. Evidence from school‐based deworming trials indicates that albendazole and mebendazole alone often produce inconsistent cognitive benefits, particularly in settings where reinfection risk persists due to inadequate sanitation. This highlights the importance of coupling deworming programs with water, sanitation, and hygiene (WASH) initiatives to sustain reductions in parasite burden and translate them into long‐term improvements in attention, memory, and academic performance.

Similarly, findings from schistosomiasis interventions with praziquantel demonstrate that nutritional status influences cognitive recovery, suggesting that school feeding and micronutrient supplementation should be incorporated into treatment campaigns. Malaria chemoprevention with artemisinin‐based combination therapies (ACTs) has shown stronger cognitive benefits than older regimens, with outcomes further enhanced when combined with vector control measures such as insecticide‐treated nets, reinforcing the need for multilayered strategies. These recommendations are consistent with the WHO's NTD Roadmap (2021–2030) (WHO 2020), which advocates for cross‐sectoral approaches to accelerate progress against neglected tropical diseases. Embedding parasite control within broader child health and education frameworks, including WASH, nutrition, and school attendance monitoring, ensures that interventions address both biomedical and social determinants of learning outcomes. Situating school‐based drug administration within this global policy framework enables national governments to strengthen accountability, mobilize donor support, and ensure that parasite control contributes directly to human capital development and equity objectives.

Finally, investment in standardized cognitive assessment frameworks is critical. Sensitive instruments such as the Stroop Test, Trail Making Test, and Digit Span consistently detect subtle improvements, whereas broader IQ measures often fail to capture shorter changes. Incorporating these tools into program evaluation would allow policymakers to monitor both immediate and long‐term effects, thereby enhancing accountability and ensuring comparability across regions.

## 4 Conclusion

This systematic review highlights the substantial adverse effects of parasitic infection, including *Schistosoma* spp., soil‐transmitted helminths, *Plasmodium* spp., and other parasites such as tungiasis, tapeworms, liver flukes, and pinworms, on the neurocognitive and educational outcomes of school‐aged children in endemic regions. Evidence consistently demonstrates deficits across several cognitive and educational domains, including memory, attention, and reaction time, executive functioning, and school performance. The variability in outcomes is attributable to differences in parasite species, study design, drug regimen, and follow‐up duration.

Although antiparasitic interventions have proven effective in reducing infection prevalence, their impact on cognitive recovery remains variable, reflecting the complex interactions between biological, nutritional, and environmental determinants of child development. Preventive chemotherapy with agents such as praziquantel, albendazole, mebendazole, and artemisinin‐based therapies has proven effective in lowering infection prevalence; however, the associated cognitive and academic gains remain inconsistent.

More sensitive neuropsychological assessments, including the Stroop Test, Trail Making Test, and Digit Span, were better able to capture subtle improvements, whereas broader intelligence measures often failed to detect short‐term changes. These findings emphasize the need for standardized and culturally validated cognitive assessment frameworks to ensure accurate evaluation of intervention outcomes.

### 4.1 Strengths and Limitations

This review is characterized by its comprehensive scope, extending beyond soil‐transmitted helminths to include *Schistosoma* and *Plasmodium* species, thereby reflecting the epidemiological reality of polyparasitism in endemic regions. The incorporation of both randomized controlled trials and observational studies enhances ecological validity while permitting causal inference where feasible. Furthermore, the structured classification of neurocognitive outcomes into four domains: memory, attention/reaction time, executive function/intelligence, and school performance, improves interpretability and facilitates cross‐study comparability. The wide geographic coverage, with evidence drawn from more than 16 countries, strengthens the generalizability of findings and captures heterogeneity in infection types, intensities, and assessment methodologies.

Despite these strengths, several limitations warrant consideration. The predominance of observational studies restricts causal inference, even when quality assessments are applied. Variability in cognitive assessment tools complicates synthesis, and the allocation of instruments to specific domains occasionally requires subjective judgment, particularly when measures span multiple cognitive abilities. Moreover, many studies failed to adequately control confounding factors such as nutritional status, malaria co‐infection, socioeconomic conditions, and school quality. These methodological and contextual limitations may obscure the true impact of parasitic infections on child development and reduce the precision of the conclusions drawn.

### 4.2 Remaining Gaps and Recommendations for Future Research

Despite the comprehensive scope of this review, several key gaps in the existing literature warrant further investigation. The predominance of observational studies limits the ability to establish definitive causal relationships, highlighting the need to design randomized controlled trials incorporating robust cognitive outcome measures. Additionally, the lack of standardized and culturally validated neuropsychological assessment tools for African children hampers direct study comparisons. Future research should focus on developing and implementing universally accepted cognitive assessment methodologies to enhance data accuracy and comparability.

### 4.3 Recommendations

1. Design randomized controlled trials incorporating robust cognitive outcome measures.
2. Develop and implement universally accepted cognitive assessment methodologies to enhance data accuracy and comparability.
3. Adequately account for confounding variables such as nutritional deficiencies, malaria co‐infection, socioeconomic conditions, and educational quality.
4. Adopt integrated research approaches examining multiple health and developmental factors concurrently to provide deeper insights into compounded effects.
5. Conduct longitudinal studies with extended follow‐up periods to track long‐term cognitive trajectories and assess clearer dose–response relationships.
6. Investigate the molecular and neurobiological mechanisms underlying the impact of different parasites on brain development, beyond the general effects of inflammation and anemia.
7. Undertake implementation research to optimize intervention strategies that integrate deworming and malaria control with nutritional support, sanitation improvements, and educational programs.
8. Develop comprehensive, multisectoral public health initiatives to ensure sustainable cognitive and educational benefits for affected children.

## Author Contributions

Conceptualization—A.M.A., D.L.S., and A.B.; methodology and investigation—A.M.A., D.M.D., K.S.M., M.H.A., G.N.O., and L.K.; Supervision—D.L.S. and A.B.; Writing – original draft—A.M.A., D.M.D., K.S.M., M.H.A., G.N.O., and L.K. All authors revised the manuscript critically for important intellectual content. All authors read and agreed with the final manuscript.

## Funding

The authors have nothing to report.

## Conflicts of Interest

The authors declare no conflicts of interest.

## Consent Statement

The authors have nothing to report.

## Supporting information




**Supplementary Table**: brb371326‐sup‐0001‐SuppMat.docx

## Data Availability

No primary data were used for the research described in the article.
