# Supplementary material for: Parasitic Infections and Associated Cognitive Outcome Among School‐Aged Children in Africa: A Systematic Review
Source: Brain Behav. 2026 Mar 30;16(4):e71326. doi: 10.1002/brb3.71326 (PMC13112015; doi:10.1002/brb3.71326)
Supplement: Supplementary file 1 — Supplementary Table: brb371326‐sup‐0001‐SuppMat.docx [file BRB3-16-e71326-s001.docx]

**Search Strategy**

**PubMed**

("Parasitic Diseases"[MeSH] OR "Helminths"[MeSH] OR "Helminthiasis"[MeSH] OR

"Schistosomiasis"[MeSH] OR "Schistosoma haematobium"[MeSH] OR "Schistosoma mansoni"[MeSH] OR

"Malaria"[MeSH] OR "Plasmodium falciparum"[MeSH] OR "Toxoplasmosis"[MeSH] OR "Giardiasis"[MeSH] OR

"Neurocysticercosis"[MeSH] OR "Soil-Transmitted Helminthiasis"[MeSH] OR "Hookworm Infections"[MeSH] OR

"Ascaris lumbricoides"[MeSH]

OR "parasitic infections" OR helminths OR schistosomiasis OR malaria OR toxoplasmosis OR giardiasis OR neurocysticercosis OR "soil-transmitted helminths" OR hookworm OR Ascaris)

AND

("Child"[MeSH] OR "Child, Preschool"[MeSH] OR "Adolescent"[MeSH] OR "Students"[MeSH]

OR "school-aged children" OR "primary school children" OR "school children" OR pupils OR students)

AND

("Cognition"[MeSH] OR "Cognitive Dysfunction"[MeSH] OR "Memory"[MeSH] OR "Learning"[MeSH] OR "Attention"[MeSH]

OR "Executive Function"[MeSH] OR "Intelligence"[MeSH] OR "Reaction Time"[MeSH] OR "Neuropsychological Tests"[MeSH]

OR "Educational Status"[MeSH] OR "Academic Performance"[MeSH]

OR "cognitive performance" OR "cognitive function" OR "academic performance" OR intelligence OR IQ OR memory OR attention OR "learning ability" OR "school performance" OR "executive function")

AND

("Africa"[MeSH] OR "Africa South of the Sahara"[MeSH] OR "Africa, Eastern"[MeSH] OR "Africa, Western"[MeSH] OR "Africa, Northern"[MeSH] OR "Africa, Central"[MeSH] OR "Developing Countries"[MeSH]

OR Africa OR "Sub-Saharan Africa" OR "low-income countries" OR "developing countries")

**Scopus**

("parasitic infections" OR "helminths" OR "schistosomiasis" OR "malaria" OR "toxoplasmosis" OR "giardiasis" OR "neurocysticercosis" OR "soil-transmitted helminths" OR "hookworm" OR "Ascaris")

AND

("school-aged children" OR "primary school children" OR "school children" OR "pupils" OR "students")

AND

("cognitive performance" OR "cognitive function" OR "academic performance" OR "intelligence" OR "IQ" OR "memory" OR "attention" OR "learning ability" OR "school performance" OR "executive function")

AND

("Africa" OR "Sub-Saharan Africa" OR "low-income countries" OR "developing countries")

**Google Scholar (Advanced Search):**

**with all of the words:** impact of parasitic infection on cognitive function among school children

**with the exact phrase:** parasitic infection

**with at least one of the words:** cognitive function

**where my words occur:** anywhere in the article
